# High‐throughput microfluidic real‐time PCR as a promising tool in disease ecology

**DOI:** 10.1111/1365-2656.70088

**Published:** 2025-06-27

**Authors:** Tristan Bralet, Rachid Aaziz, Jérémy Tornos, Amandine Gamble, Augustin Clessin, Mathilde Lejeune, Clémence Galon, Lorraine Michelet, Célia Lesage, Tiphaine Jeanniard du Dot, Guillaume Desoubeaux, Muriel Guyard, Sabine Delannoy, Sara Moutailler, Karine Laroucau, Thierry Boulinier

**Affiliations:** ^1^ CEFE, CNRS, Université Montpellier, EPHE, IRD Montpellier France; ^2^ Anses, Animal Health Laboratory, Bacterial Zoonoses Unit Maisons‐Alfort France; ^3^ School of Biodiversity, One Health and Veterinary Medicine University of Glasgow Glasgow UK; ^4^ Department of Public and Ecosystem Health Cornell University Ithaca New York USA; ^5^ ANSES, INRAE, Ecole Nationale Vétérinaire d'Alfort, UMR BIPAR, Laboratoire de Santé Animale Maisons‐Alfort France; ^6^ Environmental Department Terres Australes et Antarctiques Françaises (TAAF) Saint‐Pierre La Réunion France; ^7^ Centre d'Etudes Biologiques de Chizé, Centre National de la Recherche Scientifique Villiers‐en‐Bois France; ^8^ Parasitology and Mycology Department Bretonneau Hospital Tours France; ^9^ Research Center for Respiratory Diseases Inserm UMR1100, University of Tours Tours France; ^10^ Anses, Ploufragan‐Plouzané Laboratory Unit of Hygiene and Quality of Poultry and Pork Products Ploufragan France; ^11^ Anses, Laboratory for Food Safety COLiPATH Unit & Genomics Platform IdentyPath Maisons‐Alfort France

**Keywords:** avian cholera, disease ecology, high‐throughput real‐time PCR, infectious agents, penguins, seabirds, subantarctic communities, wildlife health monitoring

## Abstract

1. Among recent advances in molecular biology for studying infectious diseases, the microfluidic high‐throughput real‐time polymerase chain reaction (Htrt PCR) has emerged as an efficient first‐line tool for the detection of a wide range of infectious agents (IA) in a host system. This technology allows large batches of samples to be screened simultaneously for tens of targeted IA by real‐time PCR. It represents a promising tool in disease ecology.
2. As a proof of concept, we present here the development steps and initial application of a microfluidic Htrt PCR system for the detection of DNA from 28 selected IA in a set of wild vertebrates. We applied this approach to 497 samples (mainly mucosal swabs and necropsy tissues) from 274 seabirds and 80 mammals from Southern Ocean islands. This ecosystem is of particular interest for disease ecology and biodiversity conservation due to the high host densities of breeding colonies, within and among which pathogens may spread rapidly. Positive samples were subsequently confirmed for each PCR system using a secondary real‐time or conventional PCR system and/or sequencing.
3. Fourteen targeted IA were detected. The approach allowed an efficient screening of host species for known seabird pathogens, including *Pasteurella multocida* (avian cholera) (9.9% [6.6–14.0] in seabirds, 18.3% [9.5–30.4] in mammals) and *Erysipelothrix amsterdamensis* (15% [11–19.8] in seabirds, 2.1% [0.4–6.1] in mammals) in cloacal and rectal swabs. Their detection on islands where they were not known previously raised conservation concerns. IA not previously known to be circulating in the system were detected at high prevalence, notably *Chlamydiaceae* on all colonies (32.1% [26.6–38] in seabirds and 21.3% [12.9–31.8] in mammals, in cloacal and rectal swabs). Prevalence and diversity of targeted IA could be compared among sites and host species, highlighting the usefulness of the approach to explore drivers of IA community dynamics, but also to identify specific hosts as potential epidemiological sentinels or reservoirs.
4. Htrt PCR is a powerful and versatile tool that can be used in disease ecology for exploring the composition of IA communities within host communities, but also for addressing other important basic and applied questions in multi‐host, multi‐pathogen systems.

## Graphical abstract

This work highlights the diversity of disease ecology questions Htrt PCR could contribute to addressing in a given host–pathogen ecosystem. This tool could be used to answer a broad set of basic and applied questions in animal ecology, notably by providing a way to address issues involving communities of hosts and communities of pathogens.

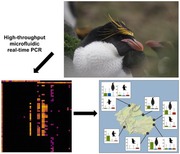

## Résumé

1. Parmi les progrès récents de la biologie moléculaire dans l'étude des maladies infectieuses, la PCR en temps réel microfluidique à haut débit (Htrt PCR) apparaît comme un outil de première intention efficace pour la détection d'un large panel d'agents infectieux (IA) dans un système donné. Cette technologie permet de tester simultanément un grand nombre d'échantillons pour des dizaines d'agents infectieux ciblés par PCR en temps réel et représente donc un outil prometteur en écologie des maladies infectieuses.
2. Comme preuve de concept, nous présentons ici la mise en place et l'application d'un système Htrt PCR pour la détection de 28 IA à ADN dans des populations de vertébrés sauvages. Cette approche a été appliquée à 497 échantillons (écouvillons de cloacaux et tissus d'autopsie) provenant de 368 oiseaux marins et mammifères des îles subantarctiques. Cet écosystème présente un intérêt particulier en écologie des maladies infectieuse en raison notamment des fortes densités des colonies au sein desquelles la circulation des IA peut être importante. Tous les échantillons positifs en Htrt PCR ont été confirmés secondairement par des PCR en temps réel ou classiques et/ou par séquençage.
3. Quatorze des IA ciblés ont été détectés. L'approche a permis de rechercher efficacement certains IA connus pour être associés à des mortalités d'oiseaux marins, comme l'agent du choléra aviaire (*Pasteurella multocida*) (9,9% [6,6‐14,0] chez les oiseaux marins et 18,3% [9,5‐30,4] chez les mammifères) ou la bactérie *Erysipelothrix amsterdamensis* (15% [11–19,8] chez les oiseaux marins et 2,1% [0,4‐6,1] chez les mammifères). Leur détection sur des îles jusqu'à présent considérées comme indemnes soulève avec de fortes implications dans la conservation des espèces. Certains IA, dont la circulation dans ce système n'était pas connue auparavant, ont été détectées avec de fortes prévalences, comme la famille des *Chlamydiaceae* sur toutes les colonies (32,1% [26,6–38] chez les oiseaux marins et 21,3% [12,9‐31,8] chez les mammifères). La prévalence et la diversité des IA ciblés ont pu être comparées entre les sites et les espèces hôtes, soulignant l'utilité de l'approche dans l'exploration des dynamiques des communautés d'IA, ainsi que dans l'identification de potentielles espèces sentinelles épidémiologiques ou réservoirs.
4. La PCR Htrt semble être un outil intéressant pour l'écologie des maladies infectieuses, permettant d'explorer la composition des communautés d'IA au sein des communautés d'hôtes, tout en abordant des questions fondamentales et/ou appliquées sur les systèmes multi‐hôtes et multi‐pathogènes.

## 1 INTRODUCTION

Emerging infectious diseases are an increasing concern in many ecosystems due to global change (Mahon et al., 2024). Exploring eco‐epidemiological processes in animal populations and communities is therefore important not only for basic science but also for biological conservation, global health and socio‐economic issues (Dobson et al., 2020; Gupta et al., 2020; Manlove et al., 2016). Understanding infectious agents (IA) dynamics in wild communities requires the integration of various types of data, starting with their detection in a host. This is the first step in identifying susceptible and competent host species and exposed populations (Tompkins et al., 2011). Combined with insights into IA pathogenicity, host immune responses and host ecology, these data can reveal key processes in IA dynamics within host communities, such as the role of hosts in IA maintenance and spread (Vicente & VerCauteren, 2019; Worsley‐Tonks et al., 2020), the spatial and cross‐species transmission of IA (Behdenna et al., 2019) and the potential risks to human health (Barroso et al., 2023; Eby et al., 2023).

Host exposure to an IA can be assessed through indirect or direct approaches. Indirect approaches include the detection of host antibodies specific to an IA following the development of a humoral immune response to exposure (Tizard, 2013). Direct approaches include microscopic observation or culture of the IA, but most commonly rely on the detection of specific nucleic acid sequences (Coutlée & Franco, 2011), typically using polymerase chain reaction (PCR). As a targeted approach, PCR detects the presence or absence of a specific genetic segment and thus requires prior knowledge of the genetic sequence of the IA.

When targeting several IA, multiplex PCR (Sint et al., 2012) or simultaneous multiple singleplex PCR enables rapid and targeted analyses, with the possibility of simultaneously analysing a large number of small volume samples for several IA. Among these approaches, the microfluidic high‐throughput real‐time PCR (Htrt PCR) uses a chamber array of microchips capable of thousands of parallel singleplex real‐time PCR reactions at the nanolitre scale (Liu et al., 2003), allowing rapid screening of tens of samples for a broad panel of IA (Michelet et al., 2014).

So far, Htrt PCR has notably been used for human disease surveillance, health assessments of domestic or wild animals and for the detection of antimicrobial resistance genes (Bakhshi et al., 2020; Croville et al., 2018; Delannoy et al., 2023; Malmsten et al., 2019; Michelet et al., 2014; Moutailler et al., 2019). Given its properties and previous applications, we believed that this approach could be a promising tool in disease ecology. Here, we outline the steps required to adapt the tool to a new system and illustrate its application to a multipathogens multivertebrate hosts system as a proof of concept. Specifically, we developed and applied an Htrt PCR that simultaneously runs 48 PCR on 48 samples to study IA occurrence patterns in vertebrate host communities breeding on Southern Ocean islands. Several species in these communities are threatened by human activities (Dias et al., 2019; Grémillet & Boulinier, 2009), and some of them are known to host IA that can cause significant disease outbreaks, such as highly pathogenic avian influenza (see e.g. Bennison et al. (2024) and Clessin et al. (2025) for details on current panzootic) or avian cholera (Jaeger et al., 2020).

The Htrt PCR tool can address various disease ecology questions, such as (1) whether pathogens already identified for being responsible for mortalities at some locations can be found circulating at other locations, (2) how IA community composition varies with host life‐history traits (we expected a lower IA prevalence and diversity in avian mesopredators (e.g. albatrosses, penguins, burrowing petrels) compared to apex avian scavengers and predators (e.g. skuas, giant petrels) given the latter's higher trophic position and presumed repeated exposure on infected carrion), (3) the potential for avian scavengers and predators to serve as sentinel species (as described in Halliday et al., 2007) and (4) the role of introduced mammals in the spread and maintenance of IA in native seabird populations (in particular, we hypothesized that introduced mammals on Amsterdam Island could act as reservoirs for pathogens such as the agent of avian cholera affecting local seabirds). Such an approach could be especially useful for projects involving large‐scale sampling of wild host communities, given increased awareness about the importance of the emergence of pathogens in wildlife.

## 2 MATERIALS AND METHODS

### 2.1 Selection of IA of interest

From our ongoing work on Southern Ocean islands ecosystems and an extensive literature review, we identified a pool of bacterial, fungal and protozoal IA of potential interest to target. We included *Pasteurella multocida*, the causative agent of avian cholera, and *Erysipelothrix* spp., both of which have been identified as potential threats to some albatross and penguin populations (Cooper et al., 2009; Zhong et al., 2024). We also considered IA documented to occur in subantarctic areas and in similar seabird systems, such as the North Atlantic or western Indian Oceans. Reviews by Barratclough et al. (2023), Bonnedahl et al. (2005), Grimaldi et al. (2015), Khan et al. (2019) and Uhart et al. (2018) were especially useful. IA suspected to circulate through the passive epidemiological surveillance programme conducted in the French Southern Territories since 2017 were also included (Boulinier et al., 2016). For each IA, we chose to target either a single IA species or a broader taxonomic level, typically at the genus level. This choice was motivated by biological and methodological factors: For some genera, all members are pathogenic and thus relevant to study as a whole, whereas for other genera, the genetic variability within and between species is not sufficiently documented to allow the design of PCR sets that would ensure species‐level specificity.

### 2.2 Design and validation of primers and probes for the detection of IA

We specifically designed PCR sets to meet the constraints of microfluidic technology, such as ensuring that all primers have the same melting temperature as all singleplex PCR are run with the same cycling conditions in the microfluidic array (see criteria in Supporting Information S1). We either adapted existing PCR sets or newly designed them from IA sequences available on GenBank (nih.gov). For some genera, multiple primer/probe pairs were required to ensure the detection of all key species within the targeted genus under consideration.

Primers, probes and synthetic control oligonucleotides were synthetized by Eurofins (MWG Operon, Germany) and PCR sets were individually validated by real‐time PCR using positive controls (Supporting Information S3). For primers and probe sets validation tests, real‐time PCR were performed in a final volume of 20 μL on a ViiA7 instrument (Applied Biosystems, Waltham, MA, USA) as described in Aaziz et al. (2022).

### 2.3 Sample collection

Samples were collected in the French Southern Territories in the Southern Indian Ocean. The main fieldwork was conducted on Possession Island (Crozet archipelago, 46°24′41″ S, 51°45′22″ E). This 150 km^2^ island hosts high densities of seabirds and is a critical breeding place for subantarctic seabird communities (Delord et al., 2014).

We collected samples from nine native bird species at different sites on Possession Island in December 2021: five avian mesopredators (king penguin *Aptenodytes patagonicus*, macaroni penguin *Eudyptes chrysolophus*, rockhopper penguin *Eudyptes chrysocome*, gentoo penguin *Pygoscelis papua* and white‐chinned petrel *Procellaria aequinoctialis*) and four apex predators and scavengers (brown skua *Stercorarius antarcticus*, Northern and Southern giant petrels *Macronectes halli* and *M. giganteus*, lesser sheathbill *Chionis minor*). Field campaigns took place during the early austral summer, that is, the early breeding season (egg‐laying and first hatch) for most species, or the late chick rearing period for gentoo penguins and giant petrels. All individuals were apparently healthy adults at the time of capture. Penguins were captured by hand, while flying birds were caught using a noose pole or a hook. For all species, cloacal and/or oral swabs were collected. Sex was determined for some individuals, but the breeding status was not assessed.

In addition, we sampled healthy individuals from two native mammal species: Subantarctic fur seals (*Arctocephalus tropicalis*) on Amsterdam Island (37°49′33″ S, 77°33′17″ E) in 2021 (oral and rectal swabs) and Southern elephant seals (*Mirounga leonina*) on the Kerguelen Archipelago (49°15′S, 69°10′ E) in 2022 (nasal swabs) during the early breeding and the moulting season, respectively (Supporting Information S4). Additionally, two species of introduced rodents (brown rat *Rattus norvegicus* and house mouse *Mus musculus*) were sampled on Amsterdam Island. Individuals were live‐trapped, euthanised and sampled for mucosal swabs and internal tissues.

In complement, a selected set of samples were collected as part of a passive surveillance programme set up to monitor abnormal mortalities. Field necropsies were conducted systematically, resulting in samples (mucosal swabs and internal tissues) from 14 cases of abnormal mortality detected in 2021 and 2022 in Crozet and Kerguelen Islands from various species (Supporting Information S4).

Swabs were stored in Longmire buffer (Longmire et al., 1997) and tissues were stored in Longmire or RNAlater preservation buffer (Qiagen), except for the kidneys of subantarctic fur seals, which were stored in 70% ethanol. All samples were kept at cool ambient temperatures for a maximum of 7 days during fieldwork before being stored at −20°C.

### 2.4 DNA extraction

Nucleic acids were extracted from swabs and tissues using the MagMax™ CORE Nucleic Acid Purification Kit (Thermo Fisher Scientific, Courtaboeuf, France) according to the manufacturer's instructions. Before the lysis step, the swabs were vortexed for 2 min and the tissues were processed with Fastprep®‐24 5G, Lysing Matrix D (MP Biomedicals, Illkirch, France) using a Precellys® Evolution Touch instrument (Bertin Technologies, Montigny le Bretonneux, France) with three repetitions of 20 s at 6800 rpm. We proceeded with 200 μL of supernatant. After an overnight lysis step, nucleic acids were purified on 96‐well plates in 100 μL elution buffer using a KingFisher Flex extraction automat (Thermo Fisher Scientific, Courtaboeuf, France).

An endogenous extraction indicator consisting of a real‐time PCR directed against the sequence of the gene encoding for the avian and mammalian ß‐actin (ID‐VET, Grabels, France) was used to assess the success of the sample preservation and DNA extraction. Confirmation was performed on ten randomly selected wells and the results extrapolated to the whole 96‐well plate (Supporting Information S2).

### 2.5 DNA pre‐amplification

In order to increase the probability that each targeted sequence was found in each reaction well (since the DNA extract is divided into a few nanolitres in each of the well) and thus to increase the probability of detecting each IA, a single pre‐amplification step was performed prior to the Htrt PCR step. PCR fragments were pre‐amplified using the PreAmp Master Mix (Standard Biotools, CA, USA) and then diluted 1:10 in Milli‐Q ultrapure water as described in Michelet et al. (2014). This step results in a reduction of the cycle threshold value (Ct) by approximately 10 points in the subsequent PCR (Michelet et al., 2014).

### 2.6 High‐throughput real‐time PCR

Htrt PCR amplification was performed using a 48.48 dynamic array (Standard Biotools, CA, USA) on a BioMark real‐time PCR instrument (Standard Biotools, CA, USA).

In a first step, each assay mix and sample mix was added to the array in individual wells prior to thermal cycling, resulting in 2304 individual reactions in a final volume of 9 nL with the cycling parameters previously described (Michelet et al., 2014). Data were analysed using Fluidigm real‐time PCR analysis software to obtain Ct values.

One negative water control and two positive controls, consisting of a mixture of all positive DNA controls divided into two wells, were included per array. To determine whether factors present in the DNA sample could inhibit the real‐time PCR, a plasmid encoding enhanced green fluorescent protein (pEGFP) was added to one of the real‐time PCR mixes containing primers and probes directed to its DNA sequence (Supporting Information S1). The correct amplification of pEGFP was thus verified for each sample. Real‐time PCR sets from IA of particular interest were used in duplicate (e.g. *P. multocida*; Jaeger et al., 2020). To ensure specificity and absence of cross reaction between all PCR sets, an Htrt PCR run was performed on all positive controls (Supporting Information S5). All Ct values >30 (equivalent to the commonly used threshold of 40 in conventional real‐time PCR) were considered negative.

### 2.7 Validation of results

For each IA and for a selection of samples that tested positive for the IA of interest, a secondary PCR (real‐time PCR and/or endpoint PCR) targeting different genes or regions than those targeted by the Htrt PCR was performed to confirm the presence of the IA (Supporting Information S6). Amplicons of particular interest were sequenced (Eurofins) and assembled (BioEdit software, Ibis Biosciences, Carlsbad). The resulting sequences were compared with published sequences in the GenBank database using online BLAST software.

### 2.8 Data analysis

Analyses were performed using R v4.3.3 (R Core Team, 2021). Prevalences and 95% Clopper–Pearson confidence intervals were those calculated from cloacal/rectal swab data (except for elephant seals, which had nasal swabs only). IA diversity was estimated by calculating specific richness and Shannon index (Fath, 2018) using the ‘vegan’ package. To avoid redundancy, we considered only the results of PCR sets targeting the broadest taxonomic ranks for each IA, acknowledging inequality in taxonomic ranks between taxa. This included the following 18 taxa: *Aspergillus fumigatus*, *Fusarium* spp., Mucorales, *Toxoplasma gondii*, *Borrelia* spp., *Coxiella burnetii*, *Brucella* spp., *Campylobacter coli*, *C. jejuni*, *C. lari*, *Leptopsira* spp., *Chlamydiaceae*, *Erysipelothrix amsterdamensis*, *E. rhusiopathiae*, *Mycobacterium* spp., *P. multocida*, *Salmonella* spp. and *Yersinia* spp.

Generalized linear models with Poisson distributions were implemented using ‘ggeffect’ and ‘Ttest’ packages to test Shannon index and specific richness in seabirds as a function of species in combination with site as random effects (Figure 4). Maps were generated with the connectors ©Stadia Maps on ©OpenStreetMap (https://tile.openstreetmap.org/).

### 2.9 Ethical approval

The sampling of live animals in the French Southern and Antarctic Lands was granted by the French Ministry of Research (APAFIS #31773‐2019112519421390 v4) and TAAF administration (A‐2021‐55) after evaluation by Comité Regional d'Ethique CREA34, Comité de l'Environnement Polaire and Conseil National de Protection de la Nature.

## 3 RESULTS

### 3.1 Development of the Htrt PCR for the detection of selected IA

Based on a literature review, we selected 26 bacterial taxa, 3 fungal taxa and 1 protozoan species relevant to this study. The initial Htrt PCR panel consisted of 30 primers/probe sets targeting these IA (Supporting Information S1), with some targets duplicated and including an internal PCR control. Twenty‐eight of the PCR sets gave positive results only for their respective positive controls. The sets for *Salmonella enterica* serovar Typhimurium and Enteritidis showed cross‐reactivity; hence, their results were not considered.

### 3.2 Methodological implementation and confirmation of the Htrt PCR results

**FIGURE 1:**
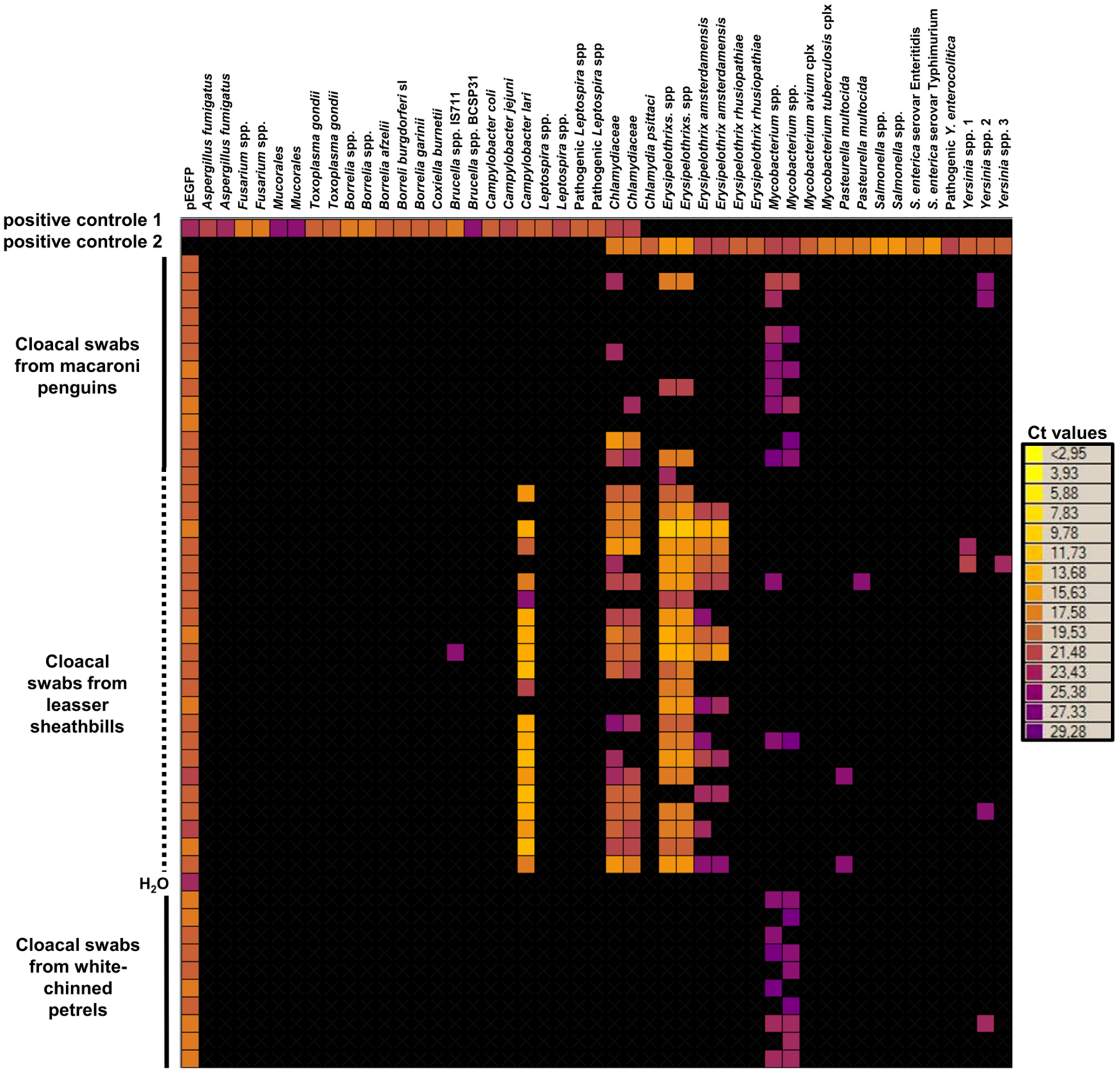
Screening of 45 seabird cloacal swabs using an Htrt PCR targeting 30 infectious agents on a 48.48 chip. Rows represent individual samples tested and columns correspond to the targets of each primer/probe set. Each square corresponds to a single real‐time PCR reaction. Ct values for each reaction are indicated in colour. All Ct values >30 were considered negative (dark square). A negative control (water) and a positive control consisting of a pool of positive controls for each PCR system, distributed in two wells, were included in each run.

A total of 497 swabs from 274 seabirds and 80 mammals and 66 samples from 14 cases of unusual mortality events were tested using the Htrt PCR. An example of the output of the results is shown in Figure 1 and a detailed description of the results is available in the Supporting Information S4.

For 11 IA, all field samples tested negative or could not be confirmed with the secondary PCR and were therefore considered negative. These included fungi (*A. fumigatus* and *Fusarium* spp.), protozoa (*T. gondi*), tick‐borne bacteria (*Borrelia* spp., the three *Borrelia* species and *C. burnetti*), mammalian‐associated bacteria (*Brucella* spp., *C. coli*, pathogenic *Leptospira*, *Mycobacterium tuberculosis* complex) and avian‐associated bacteria (*C. psittaci*, pathogenic *Y. enterocolitica*, *Mycobacterium avium* complex). Thirteen IA gave positive results that could be confirmed, including Mucorales, *Chlamydiaceae*, *Leptospira* spp., *Mycobacterium* spp., *Salmonella* spp. and *C. lari*, *C. jejuni*, *E. amsterdamensis*, *E. rhusiopathiae*, *P. multocida* and three designs for the detection of *Yersinia* spp. Unexpectedly, the primers designed for *E. amsterdamensis* amplified a sequence from a newly proposed species *E. enhydrae*, initially detected in Southern sea otters (Chang et al., 2024). Given the high genetic similarity between these two species and the limited knowledge of their genetic variability, we have chosen not to distinguish them as separate taxa and here considered them together under the *E. amsterdamensis* complex. The primers designed for *Erysipelothrix* spp. were excluded due to the lack of specificity observed when tested on this panel of samples.

For the biological analysis and interpretation of the field samples results, we used only the results from the 13 validated PCR designs described above. Given the consistent performance of the primers in the dilution test, the systematic checks for PCR inhibitors during the assays and the use of most sets in duplicate, we set the sensitivity at 95%.

### 3.3 Addressing disease ecology questions

Of the 563 samples tested, 417 were positive for at least one IA. Fourteen IA taxa were detected at least once, 11 in at least 10 samples and 4 IA were detected in more than 10% of all samples (in descending order of frequency: *Mycobacterium* spp., *Chlamydiaceae* family, *P. multocida* and *C. lari*). The number of positive samples for each IA and additional identifications by single gene sequencing are shown in Supporting Information S6 and S7.

#### 3.3.1 Distribution range of particular IA of conservation or zoonotic interest

**FIGURE 2:**
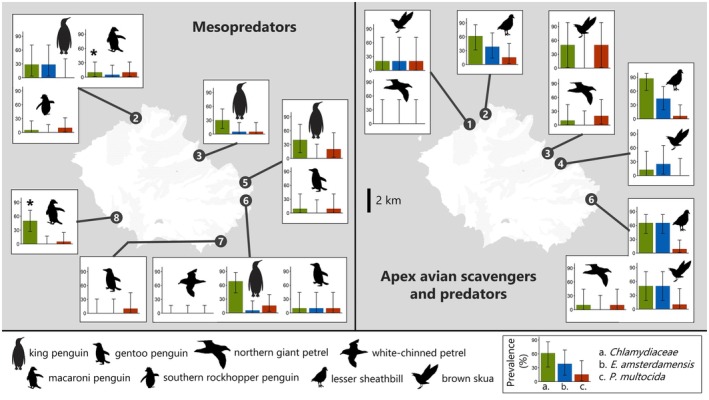
Epidemiological map for Possession Island (Crozet Archipelago) showing prevalences of IA detected in cloacal swabs using a Htrt PCR in avian mesopredators (left) and avian apex predators and scavengers (right). Three IA of particular interest are represented: *Chlamydiaceae* family (green), *Erysipelothrix amsterdamensis* complex (blue) and *Pasteurella multocida* (red). Eight sites were sampled on the island (Supporting Information S8).

**FIGURE 3:**
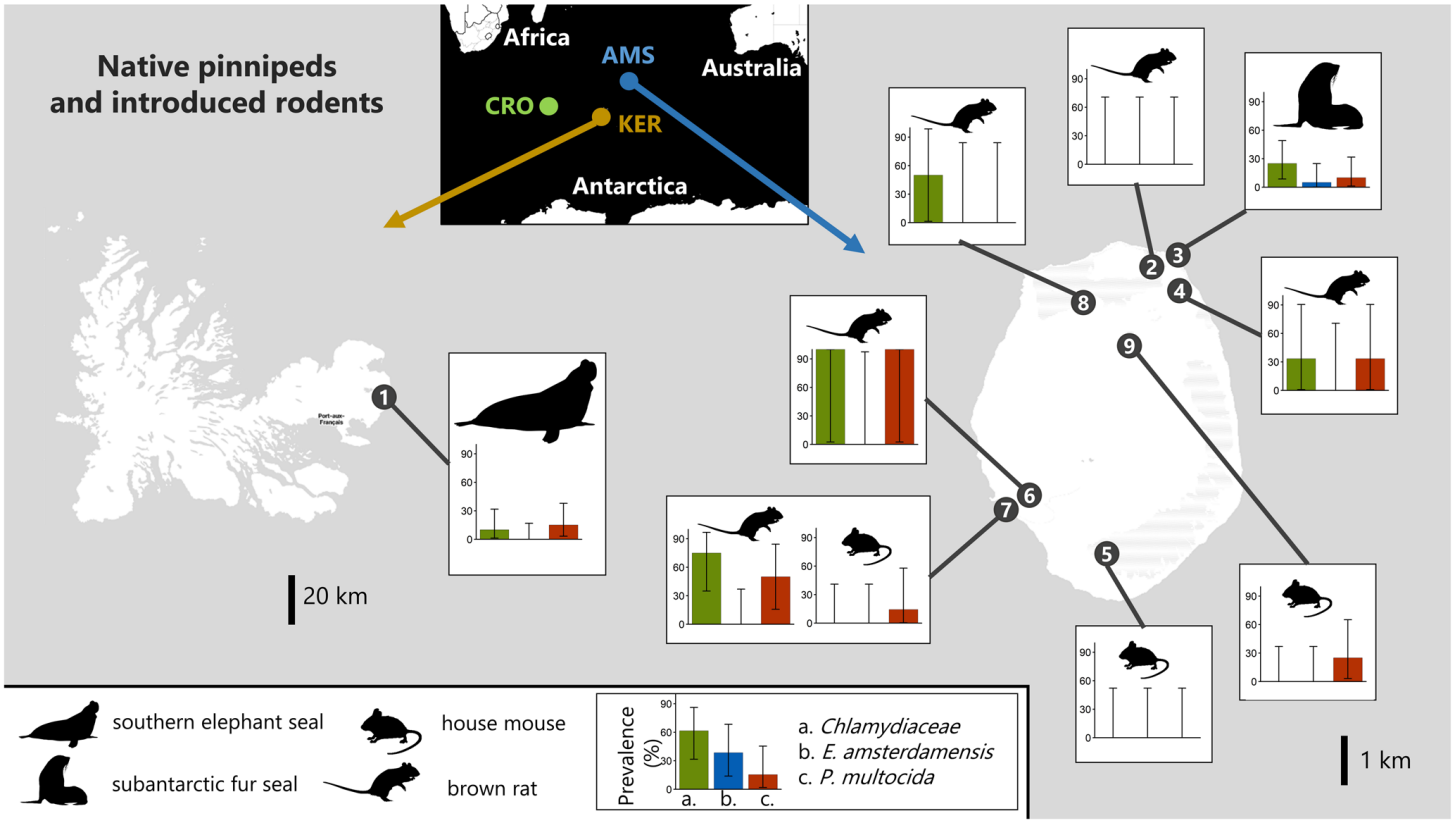
Prevalence of IA determined by Htrt PCR in mammals from two remote subantarctic territories: The Kerguelen Archipelago (left) and Amsterdam Island (right). Three IA of particular interest are represented: *Chlamydiaceae* family (green), *Erysipelothrix amsterdamensis* complex (blue) and *Pasteurella multocida* (red). The sampling sites included are outlined in Supporting information S8.

The results confirmed the predictions and hypotheses we had made for IA of particular interest in the subantarctic system. *P. multocida*, known for causing avian cholera outbreaks in seabirds on Amsterdam Island, was expected to be found in other subantarctic regions. Its presence was confirmed on the three islands, with an average of 9.9% [6.6–14.0] in seabirds on Crozet and 18.3% [9.5–30.4] in mammals on Kerguelen and Amsterdam (Figures 2 and 3). The *E. amsterdamensis* complex, associated with seabird mortalities on Amsterdam Island, was also detected in seabirds from the Crozet Archipelago with a mean prevalence of 15% [11–19.8] (4.1% [1.5–8.7] in penguins and 32.7% [23.9–42.5] in apex avian scavengers and predators). The complex was also found in mammals from Amsterdam Island, with a mean of 2.5% [0.1–13.2] in introduced mammals and 5% [0.1–24.9] in fur seals (Figures 2 and 3). Conversely, *E. rhusiopathiae* was only detected in subantarctic fur seals.

The *Chlamydiaceae* taxon, expected to be of high ecological relevance due to its wide distribution in birds, was the second most common IA, infecting 24.0% [17.7–31.2] of avian mesopredators, 44.9% [35.2–54.8] of apex avian scavengers and predators and 25% [14.7–37.9] of mammals. In particular, two samples were found to belong to the newly described genus *Chlamydiifrater* by 16S rRNA gene sequencing (Vorimore et al., 2021), but PCR analyses for the only two described species of this genus (*Chlamydiifrater phoenicopteri* and *C. volucris*) were negative, suggesting a new, as yet undescribed species.

#### 3.3.2 Detection of IA with little a priori knowledge

Among the enteric pathogens commonly found in birds, *C. lari* was the most common (21.9% [17.2–27.3] of the birds), followed by *Yersinia* spp. (12.0% [8.44–16.5] of the birds and 4.0% [1.5–8.5] of the mammals) and *Salmonella* spp., which was surprisingly only found in Subantarctic fur seals (*n* = 10/20) and in no birds (Supporting Information S8). Some unexpected results were obtained, particularly with regard to the host range of certain IA. In particular, out of the 12 samples that tested positive for non‐pathogenic *Leptospira* spp., five were from gentoo penguins. Finally, *Mycobacterium* spp. was the most commonly detected IA (32% [28.1–36] of samples), but the identification by sequencing revealed mainly environmental species and no pathogenic or zoonotic species from the *M*. *avium* or *M*. *tuberculosis* complexes (Supporting Information S7).

#### 3.3.3 Diversity of infectious agents

**FIGURE 4:**
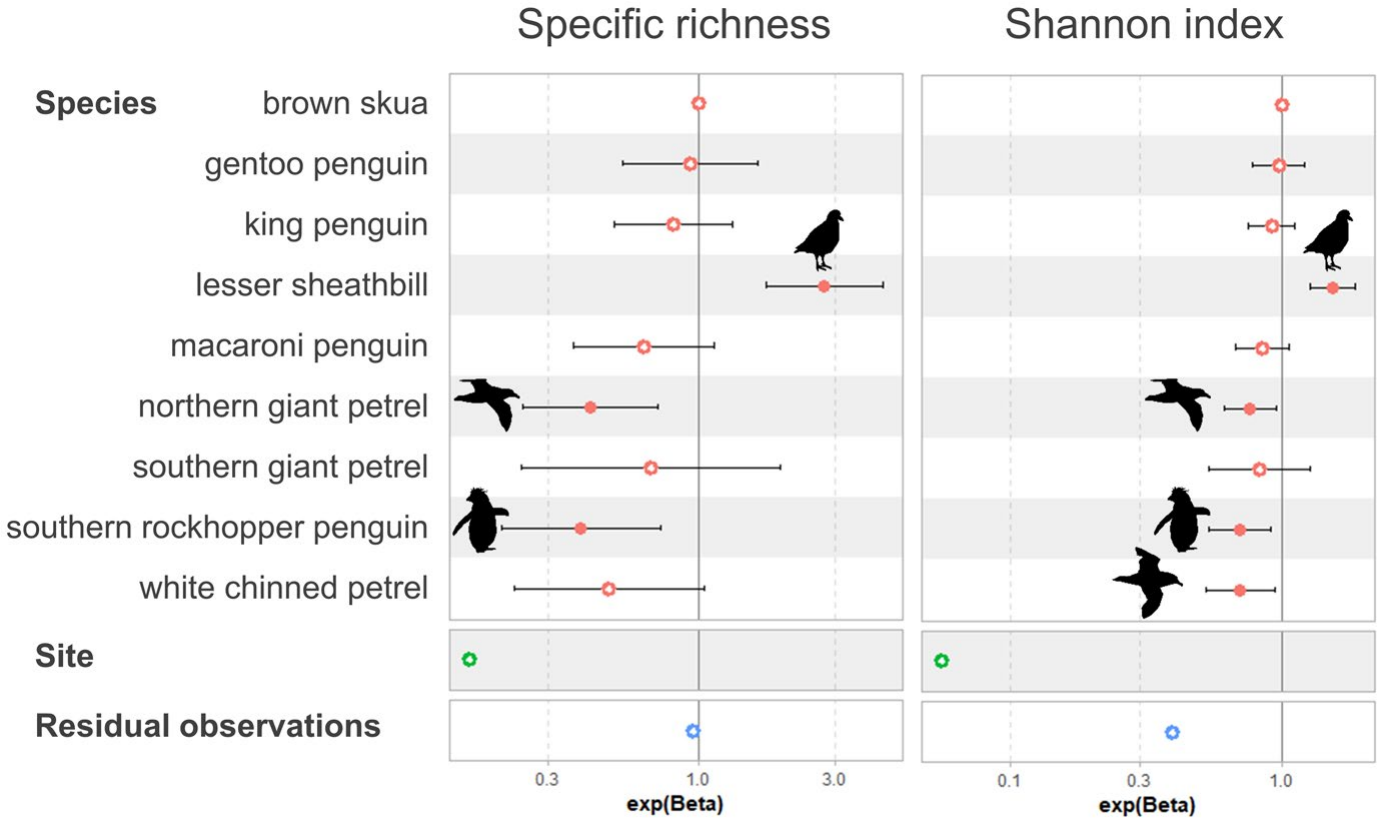
Variability in infectious agent diversity (odd ratios for specific richness and Shannon index) among seabird species. Results from GLM modelling with sampling site as a random effect (see Supporting information S9 for model summary and post hoc analyses). Solid circles represent significant terms (*p*‐value below 5%).

The richness of the number of IA found per species at a given site ranged from 0 to 6, with a median of 2 for mammals and seabirds. Nevertheless, the list of species was relatively similar. In particular, the specific richness and the Shannon index were significantly higher in the lesser sheathbill (Figure 4), highlighting the high exposure of this species to pathogens within the epidemiological system and the potential interest of using it as a sentinel species.

## 4 DISCUSSION

Understanding the patterns and drivers of infectious disease dynamics in ecosystems is a fundamental challenge. In this present work, we show how an Htrt PCR approach can provide an efficient tool for investigating multiple disease ecology questions within a given system. The key advantage of this method is its ability to simultaneously detect a large number of infectious agents in numerous samples, using minimal sample material.

### 4.1 Implementation, cost and versatility of an efficient screening tool

The time required to set up the Htrt PCR assay is an important factor to consider during its development and is primarily dependent on the availability of existing PCR systems. In our case, we for example borrowed systems developed by Michelet et al. (2014) for tick‐borne IA. For novel targets, set‐up involves literature review, adaptation or design of PCR‐based assays (Supporting information S2), testing with positive controls and ensuring no cross‐reactivity between PCR systems (Supporting information S5). The design or adaptation of the 17 systems (11 adaptations, 6 new designs) presented in this study took about 4 months. Once implemented, four to five batches of 45 DNA samples can be tested per day on a 48 × 48 array on a single BioMark real‐time PCR instrument. From a cost perspective, each batch costs approximately €125 for pre‐amplification and €350 for Htrt PCR, which equates to approximately €0.30 per IA tested per sample (excluding DNA extraction and initial purchase of PCR primers/probe sets).

A major advantage of Htrt PCR is its versatility. The system can be easily adapted to specific eco‐epidemiological questions by combining previously described PCR systems and/or developing new ones. For example, it could be extended to monitor antibiotic resistance genes in seabirds and their environment in addition to IA detection using PCR sets targeting resistance genes (Delannoy et al., 2023; Guardia et al., 2024). Given the potential for shared pathogens among vertebrate host communities of mobile species, the system we set up in this study could, for example, allow relatively rapid application of the approach to communities of migratory passerines or seabirds from the northern hemisphere.

### 4.2 Optimization of the method

High sensitivity (95%) and a probability of detection of 1 were assumed for the Htrt PCR method developed in this study, as previously reported (Michelet et al., 2014). Sensitivities for specific IA can be estimated by calculating the proportion of false negatives obtained using duplicate designs (number of positive samples divided by the total number of samples that were positive at least once) and assuming no false positives due to the validation steps following Htrt PCR. These estimates were systematically underestimated by a factor of 1.5 for *Chlamydiaceae* and *E. amsterdamensis* and up to 5.3 for *P. multocida*, probably due to the low IA load of the swabs (likely to contain a limited amount of material), resulting in high Ct values close to the detection thresholds (e.g. corresponding to an average Ct of 34 for *P. multocida* using conventional real‐time PCR; data not shown).

Given the small sample size in this exploratory study, further estimation was not performed. However, apparent sensitivity was estimated for each PCR system using dilution ranges of positive controls (see Supporting Information S10), showing as expected a strong effect of dilution on the probability to detect the DNA of the bacteria. Estimates of sensitivities and thresholds of each PCR set could be refined by comparing results on such a dilution range and/or even across samples by running models such as patch occupancy models (McClintock et al., 2010). Gómez‐Díaz et al. (2010) illustrate this for the detection of *Borrelia* spp. DNA by PCR in seabird ticks.

The pre‐amplification step, required due to the limited volume of DNA extract, prevents a direct correlation between Ct values and IA load. However, accurate quantification of infection load can be essential for studies in disease ecology (Wilber et al., 2017). A potential workaround would be to use a PCR assay targeting universal genetic sequences (e.g. 16S rRNA gene for bacteria) as a proxy for total DNA content, allowing normalization of Ct values for a semi‐quantitative estimation of each IA load.

### 4.3 Potential extension

Htrt PCR can be adapted to RNA viruses by including a reverse transcription step, as shown in a similar approach developed for tick‐borne viruses (Moutailler et al., 2019). This may be relevant for screening high impact pathogens such as influenza viruses, coronaviruses, flaviviruses or paramyxoviruses (Woolhouse et al., 2013). DNA viruses were not included here due to limitations in primer design for family detection (see Supporting information S1), but targeted assays using strain‐specific primers could be developed, particularly for Poxviridae and Herpesviridae, which have been linked to seabird mortality (Niemeyer et al., 2017; Schoombie et al., 2018).

### 4.4 Complementarity with other approaches

Because Htrt PCR detects nucleic acids, it provides a snapshot of host exposure to infectious agents but does not confirm active infection or pathogen viability. This limitation should be taken into account when interpreting ecological or epidemiological patterns, and may warrant complementary approaches such as bacterial culture or serological surveys to provide a more robust assessment of infection status.

Htrt PCR can be seen as an approach complementary to metagenomic approaches—metabarcoding and shotgun sequencing—which allow untargeted, retrospective detection of a potentially unlimited number of specific IA through computational analyses (Bergner et al., 2019). The sensitivity and contribution of each method have been extensively compared, particularly in environmental DNA studies (Harper et al., 2018; Johnson et al., 2024). While metagenomic methods have demonstrated equivalent or higher sensitivity, they often require large amounts of nucleic acids for sequencing and extensive data processing, which is costly (Ostaizka et al., 2023). While metagenomics is well suited for broad exploratory studies of IA diversity (Leclaire et al., 2023), Htrt PCR is particularly advantageous for hypothesis‐driven research (Yoccoz et al., 2001), where the appropriate IA panel is selected based on known or suspected host–parasite associations. Its scalability and minimal DNA input requirements make it a powerful and practical tool for large‐scale ecological studies aimed at identifying key host–IA interactions in diverse wildlife communities.

### 4.5 Answering questions of relevance for disease ecology

Multiple PCR‐based approaches help to address key questions in disease ecology, both from a host and pathogen perspective. Indeed, such approaches applied to large sample sizes allow the estimation of infection rates in different host species and over space, revealing patterns that would be difficult to detect using conventional singleplex diagnostic tools. For example, we reported a wider distribution of *P. multocida* than previously recognized, either in terms of spatial distribution (beyond the few islands documented in previous studies; Cooper et al., 2009; Jaeger et al., 2020) or in terms of host spectrum (Smith & Carpenter, 2006). All bird species were found to be positive, as were all mammalian species, raising questions about their role as vectors or reservoirs of IA and the impact of human‐related species introductions into a given system on its epidemiological processes (Smith & Carpenter, 2006). For instance, the highest prevalences of detection of *P. multocida* in brown rats on Amsterdam Island were detected at two sites of the Entrecasteaux cliffs (6 and 7 on Figure 3), where massive die‐offs of avian cholera have been observed for yellow‐nosed albatross chicks (Jaeger et al., 2020), while much lower detection rates were found for other sites of the island.

Similarly, the detection of *E. amsterdamensis* on remote islands not previously associated with marine vertebrate mortality suggests that this pathogen may be circulating more widely in these ecosystems (Jayasinghe et al., 2021; Zhong et al., 2024). These two IA were shared between mesopredators and apex predators and scavengers (already reported for *P. multocida* for Amsterdam Island in Gamble et al. (2019)), raising questions about the role of the latter in IA circulation and their high exposure through carrion consumption (Tornos et al., unpublished). However, sample sizes were not sufficient in the current study to detect significant differences in prevalences between these two host groups.

Htrt PCR is expected to be powerful for the detection of previously unexpected host associations. For example, we report the first detection of *Leptospira* spp. in penguins. These IA are classically associated with mammals, with very rare descriptions in birds (Hagedoorn et al., 2024), raising new questions about transmission pathways and potential environmental reservoirs.

In addition, Htrt PCR used with broadly defined primers can help to detect previously unidentified species. For example, the use of a generic *Chlamydiaceae* PCR set led to the detection of putative new species closely related to the recently described genus *Chlamydiifrater* (Vorimore et al., 2021). This finding may guide further sampling and isolation efforts to better characterize these new bacteria within the *Chlamydiaceae* family in seabirds (Isaksson et al., 2015).

Finally, although the current sample sizes in our study did not allow robust assessments of co‐infections or quantify within‐ and between‐host diversity, these critical aspects of disease ecology (Fountain‐Jones et al., 2018; Silk et al., 2017; Tompkins et al., 2011) could be efficiently explored in future studies, using this scalable and adaptable Htrt PCR approach.

### 4.6 Integration of Htrt PCR in the disease ecology toolkit

Htrt PCR is a powerful tool for assessing exposure patterns to a wide range of target IA. It can be used independently or as a first‐line screening method to refine sampling strategies or complement other analytical approaches.

The seabird system provides an excellent eco‐epidemiological model for Htrt PCR screening due to the high density of breeding colonies, relatively easy access to large numbers of individuals and straightforward cross‐species sampling. However, this tool is also applicable to more complex continental ecosystems. Htrt PCR could allow identifying host species that could play an important role in eco‐epidemiological dynamics or could be used as sentinels. In our case, the higher IA diversity found in the species of the genus *Chionis*, as in Zamora et al. (2023), confirms their greater risk of exposure and consequently reinforces their potential pertinence to be used as sentinel species (see Halliday et al. (2007) for definitions) in our epidemiological system, as could be expected based on social network, behavioural and trophic analyses (carrion and faeces‐based feeding) (Burger, 1980; Olesen, 2022). Moreover, Htrt PCR could also allow efficient identification of species representing compartments of potentially low apparent epidemiological relevance (e.g. white‐chinned petrels or mice according to our results in this given system), although this will depend on the initial sampling design and effort.

This method could also facilitate addressing eco‐epidemiological questions involving predictions about IA communities across multiple hosts, by linking IA communities to host abundance and social networks, allowing the test of predictions. For example, Htrt PCR could complement studies such as that of Barroso and Gortázar (2024), which aimed to test the dilution effect hypothesis—suggesting that species‐rich communities have a lower risk of IA emergence and transmission (Keesing & Ostfeld, 2021)—by linking social network analysis at different sites with seroprevalences for several pathogens. By simultaneously targeting tens of pathogens across multiple hosts and locations, Htrt PCR could rapidly contribute to the accumulation of relevant data, enabling robust exploration of such disease ecology hypotheses across different systems.

In summary, Htrt PCR is proving to be a promising, highly effective tool for studying infectious disease dynamics within ecosystems. Its ability to simultaneously detect multiple IA in a wide range of hosts with minimal sample material provides an efficient means of understanding disease exposure patterns across species and locations. The approach is versatile, cost‐effective and scalable. It can be used to efficiently address a range of basic and applied questions in disease ecology, in particular allowing hypothesis‐driven research to be conducted while simultaneously exploring IA communities in a wide range of hosts.

## AUTHOR CONTRIBUTIONS

Thierry Boulinier, Karine Laroucau, Jérémy Tornos, Sara Moutailler, Amandine Gamble and Tristan Bralet conceived the study. Tristan Bralet, Mathilde Lejeune, Jérémy Tornos, Augustin Clessin, Tiphaine Jeanniard du Dot, Thierry Boulinier, Célia Lesage and Rachid Aaziz performed fieldwork or helped for DNA extraction. Tristan Bralet, Sara Moutailler, Rachid Aaziz, Augustin Clessin, Lorraine Michelet, Guillaume Désoubeaux, Sabine Delannoy, Karine Laroucau and Thierry Boulinier were responsible for IA‐specific PCR set development and validation. Tristan Bralet, Clémence Galon, Rachid Aaziz and Sara Moutailler performed the Htrt PCR and Tristan Bralet, Rachid Aaziz, Augustin Clessin, Lorraine Michelet, Guillaume Desoubeaux, Muriel Guyard, Sabine Delannoy and Karine Laroucau followed up molecular confirmation steps. Tristan Bralet and Jérémy Tornos carried out the statistical analyses and Tristan Bralet produced the figures. Tristan Bralet wrote the first version of the manuscript and all authors provided inputs.

## CONFLICT OF INTEREST STATEMENT

The authors declare no competing interests.

## Supporting information


**Supporting Information S1.** List of infectious agents, PCR sets (target, primers/probe sets) and positive controls.
**Supporting Information S2.** List of criteria for the design of primers/probes for Htrt PCR and reference genomes (GenBank) used for newly designed primers/probes for this study.
**Supporting Information S3.** List of reference genomes used for newly designed primers/probes for this study and origin of positive controls used for validation.
**Supporting Information S4.** (A) Simultaneously obtained prevalences by Htrt PCR (after validation step) for 25 infectious agents on samples from systematic sampling (apparently healthy individuals). (B) Qualitative results of Htrt PCR performed on necropsy material from passive surveillance (opportunistically found carrions).
**Supporting Information S5.** BioMark™ dynamyc array system specificity test (48.48 chip).
**Supporting Information S6.** Primers used to confirm the presence of infectious agent DNA in samples using qPCR or endpoint PCR followed by gene sequencing.
**Supporting Information S7.** Validation of positive Htrt PCR results by single gene sequencing (Sanger sequencing) and best similarity with deposited sequences.
**Supporting Information S8.** Epidemiological map of French Southern Territories with prevalences of three enteric IA obtained by Htrt PCR in avian mesopredators (top left) and apex avian predators and scavengers (top right) in Possession Island (Crozet Archipelago) and mammals from Kerguelen Archipelago (bottom left) and Amsterdam Island (bottom right).
**Supporting Information S9.** (A) Generalised linear models parameters and summary (Figure 4) for comparisons of specific richness and Shannon index. (B) Post hoc analyses: Pairwise comparison of specific richness (a.) and Shannon index (b.) of IA between species using non‐parametric Kruskal–Wallis and then Wilcoxon tests.
**Supporting Information S10.** Calculation of apparent sensitivity (aSe) for each PCR system of the Htrt PCR assay.

## Data Availability

Open access to the data and codes used for the analyses is available via the following InDoRES public repository: https://doi.org/10.48579/PRO/HZCSWK (Tornos et al., 2025).
