# Supplementary material for: High‐throughput microfluidic real‐time PCR as a promising tool in disease ecology
Source: J Anim Ecol. 2025 Jun 27;94(9):1625–37. doi: 10.1111/1365-2656.70088 (PMC12424280; doi:10.1111/1365-2656.70088)
Supplement: Supplementary file 1 — Supporting Information S1. List of infectious agents, PCR sets (target, primers/probe sets) and positive controls. Supporting Information S2. List of criteria for the design of primers/probes for Htrt PCR and reference genomes (GenBank) used for newly designed primers/probes for this study. Supporting Information S3. List of reference genomes used for newly designed primers/probes for this study and origin of positive controls used for validation. Supporting Information S4. (A) Simultaneously obtained prevalences by Htrt PCR (after validation step) for 25 infectious agents on samples from systematic sampling (apparently healthy individuals). (B) Qualitative results of Htrt PCR performed on necropsy material from passive surveillance (opportunistically found carrions). Supporting Information S5. BioMark™ dynamyc array system specificity test (48.48 chip). Supporting Information S6. Primers used to confirm the presence of infectious agent DNA in samples using qPCR or endpoint PCR followed by gene sequencing. Supporting Information S7. Validation of positive Htrt PCR results by single gene sequencing (Sanger sequencing) and best similarity with deposited sequences. Supporting Information S8. Epidemiological map of French Southern Territories with prevalences of three enteric IA obtained by Htrt PCR in avian mesopredators (top left) and apex avian predators and scavengers (top right) in Possession Island (Crozet Archipelago) and mammals from Kerguelen Archipelago (bottom left) and Amsterdam Island (bottom right). Supporting Information S9. (A) Generalised linear models parameters and summary (Figure 4) for comparisons of specific richness and Shannon index. (B) Post hoc analyses: Pairwise comparison of specific richness (a.) and Shannon index (b.) of IA between species using non‐parametric Kruskal–Wallis and then Wilcoxon tests. Supporting Information S10. Calculation of apparent sensitivity (aSe) for each PCR system of the Htrt PCR assay. [file JANE-94-1625-s001.docx]

**Supporting Information – S1 | List of infectious agents, PCR sets (target, primers/probe sets) and positive controls.** Certain sets were used in duplicate ***(2)***. All probes are labelled with the FAM fluorophore and BHQ1 quencher. See **Supporting Information S3** for references and details on positive controls.

| Infectious agent | Target | Name | Sequences (5’-3’) | Length (bp) | Reference | Positive control |
| --- | --- | --- | --- | --- | --- | --- |
| Fungi | | | | | | |
| *Aspergillus fumigatus (2)* | 28S rRNA | AspFum_28S_F  AspFum_28S_R  AspFum_28S_P | CCCTCGGAATGTATCACCTC CATTTACGACCATTACGCCAG FAM-CGGGGTGTCTTATAGCCGAGGGTG-BHQ1 | 110 | Adapted from Challier et al., (2004) | culture |
| Mucorales  *(2)* | 18S rRNA | Mucorales_18S_F  Mucorales_18S_R  Mucorales_18S_P | GTGGTGCATGGCCGTTCTT CTAAGGGCATCACAGACCTG FAM-AGTTCGTGGAGTGATTTGTCTGGTTAATTCC-BHQ1 | 197 | This study | culture |
| *Fusarium* spp.  *(2)* | TEF1 | Fusarium_tef1_F  Fusarium_tef1_R  Fusarium_tef1_P | ATGGGTAAGGAGGACAAGACT GTGGTCGACTTGCCAGAGT FAM-CGACGTGGCCGATGACGACGAC-BHQ1 | 71 | Adapted from Karlsson et al., (2016) | culture |
| Protozoan | | | | | | |
| *Toxoplasma gondii*  *(2)* | B1 | ToxoGondi_B1_F  ToxoGondi_B1_R  ToxoGondi_B1_P | GTGAAATGTACCTCCAGAAAAGC GGCAGCGTCTCTTCCTCTTT FAM-CACCTAGTATCGTGCGGCAATGTGC-BHQ1 | 88 | Adapted from Galvani et al., (2019) | culture |
| Bacteria | | | | | | |
| *Borrelia* spp.  *(2)* | 23S rRNA | BorreliaSp_23S_F  BorreliaSp_23S_R  BorreliaSp_23S_P | GAGTCTTAAAAGGGCGATTTAGT  CTTCAGCCTGGCCATAAATAG  FAM-AGATGTGGTAGACCCGAAGCCGAGT-BHQ1 | 73 | Michelet et al., (2014) | synthetic oligonucleotide |
| *Borrelia afzelii* | fla | BorreliaAfz_fla_F  BorreliaAfz_fla_R  BorreliaAfz_fla_P | GGAGCAAATCAAGATGAAGCAAT TGAGCACCCTCTTGAACAGG FAM-TGCAGCCTGAGCAGCTTGAGCTCC-BHQ1 | 166 |  | synthetic oligonucleotide |
| *Borrelia garinii* | rpoB | BorreliaGar_rpoB_F  BorreliaGar_rpoB_R  BorreliaGar_rpoB_P | TGGCCGAACTTACCCACAAAA88  ACATCTCTTACTTCAAATCCTGC  FAM-TCTATCTCTTGAAAGTCCCCCTGGTCC-BHQ1 | 88 |  | synthetic oligonucleotide |
| *Borreli burgdorferi* sensu lato | rpoB | BorreliaBurg_rpoB_F  BorreliaBurg_rpoB_R  BorreliaBurg_rpoB_P | GCTTACTCACAAAAGGCGTCTT GCACATCTCTTACTTCAAATCCT FAM-AATGCTCTTGGACCAGGAGGACTTTCA-BHQ1 | 83 |  | synthetic oligonucleotide |
| *Brucella* spp. | IS711 | BrucellaSp1_IS711_F  BrucellaSp1_IS711_R  BrucellaSp1_IS711_P | CGCTCGCGCGGTGGAT  GAAGCTTGCGGACAG  FAM-ACGACCAAGCTGCATGCTGTTGTCGATG-BHQ1 | 178 | Bounaadja et al., (2009) | culture |
|  | BCSP31 | BrucellaSp2_BCSP31_F  BrucellaSp2_BCSP31_R  BrucellaSp2_BCSP31_P | TCTTTGTGGGCGGCTATCC  CCGTTCGAGATGGCCAGTT  FAM-ACGGGCGCAATCT-BHQ1 | 55 |  |  |
| *Campylobacter coli* | lpxA | CampyColi_lpxA_F  CampyColi_lpxA_R  CampyColi_lpxA_P | CATCCTAGTGCTGTAATAGAAG CACCTATGGTAGTATCTGAAAG FAM-TATTCTCGCCCCTTGCTTGATAACAACCC-BHQ1 | 148 | This study | culture |
| *Campylobacter jejuni* | mapA | CampyJeju_mapA_F  CampyJeju_mapA_R  CampyJeju_mapA _P | GAGGTGTATAAATTGCGGAGC GAAGCAAGTGTTGTATTTCATGG FAM-TTTTGCTCTGCTCTGCTTTTGTGAATTATGCG-BHQ1 | 152 | This study | culture |
| *Campylobacter lari* | lpxA | CampyLari_lpxA_F  CampyLari_lpxA_R  CampyLari_lpxA_P | GAGTAAAATTCATCCAAGTGCTG GCTCCTACAAAAGAATAAGCTTC FAM-TAGTAGAAGATGGGGCTATAATAGGTGATGAAG-BHQ1 | 87 | Adapted from Cerdà-Cuéllar et al., (2019) | culture |
| *Chlamydiaceae*  *(2)* | 23S rRNA | Chlam_CH23S_F  Chlam_CH23S_R  Chlam_CH23S_P | GAAACCAGTAGCTTATAAGCGGT  CTCGCCGTTTAACTTAACTCC  FAM-CTGGCTCATCATGCAAAAGGCACGC-BHQ1 | 106 | Adapted from Ehricht et al., (2006) | synthetic oligonucleotide |
| *Chlamydia psittaci* | enoA | Cpsi_enoA_F  Cpsi_enoA_R  Cpsi_enoA_P | GTGCGTATGGGTGCTGATG  GCCTTCATCTCCAACTCCTG  FAM-TAGCGAGATGTCTATCGTTGAGCAGTTTTTTC-BHQ1 | 84 | Adapted from Aaziz et al., (2022) | culture |
| *Coxiella burnetii* | Icd | Coxburn_Icd_F  CoxBurn_Icd_R  CoxBurn_Icd_P | AGGCCCGTCCGTTATTTTACG  CGGAAAATCACCATATTCACCTT  FAM-TTCAGGCGTTTTGACCGGGCTTGGC-BHQ1 | 74 | Michelet et al., (2014) | synthetic oligonucleotide |
| *Erysipelothrix amsterdamensis*  *(2)* | 5S rRNA | ErysiAms_5S_F  ErysiAms_5S_R  ErysiAms_5S_P | TCGTCAATTTCTAATCCCTTAGC  GTCTAAAGAAAGGGTGCCTCATT  FAM-TCCCTAGCGGGTGATTTGGGAGTTG-BHQ1 | 97 | This study | culture |
| *Erysipelothrix rhusiopathiae*  *(2)* | 5S rRNA | ErysiRhu_5S_F  ErysiRhu_5S_R  ErysiRhu_5S_P | ATATGTTTTAGGTTCAGTCGTCC  TGGACAAAGTCTAACGAAAGGTT  FAM-CCTCATTTCTCTAGCAGGTGATTTGGGAG-BHQ1 | 171 | Adapted from Pal et al., (2010) | culture |
| Non-pathogenic *Leptospira* spp.  *(2)* | 16S | LeptoSp_16S_F  LeptoSp_16S_R  LeptoSp_16S_P | GGCCACAATGGAACTGAG CCCATTGAGCAAGATTCTTAAC FAM-CACGGTCCATACTCCT-BHQ1 | 70 | Richard et al., (2021) | synthetic oligonucleotide & culture |
| Pathogenic *Leptospira* spp.  *(2)* | LipL32 | LeptoPath_lipl32_F  LeptoPath_lipl32_R  LeptoPath_lipl32_P | GGATCTGTGATCAACTATTACGG GTTGGGGAAATCATACGAACTC FAM-ATGTAAAGCCAGGACAAGCGCCGGA-BHQ1 | 43 |  | synthetic oligonucleotide & culture |
| *Mycobacterium* spp.  *(2)* | atpE | MycoSpAtpE_F  MycoSpAtpE_R  MycoSpAtpE_P | CGGYGCCGGTATCGGYGA CGAAGACGAACARSGCCAT FAM-ACSGTGATGAAGAACGGBGTRAA-BHQ1 | 182 | Radomski et al., (2013) | culture |
| *Mycobacterium avium* complex | IS1245 | MycoCplx_IS1245_F  MycoCplx_IS1245_R  MycoCplx_IS1245_P | GCCGCCGAAACGATCTAC TGACCCGGTGCGCAGCTT FAM-TCG CGT CCG CGC ACG CTG TCC-BHQ1 | 177 | Michelet et al., (2018) | culture |
| *Mycobacterium tuberculosis* complex | Mpb70 | MycoTub_Mpb70_F  MycoTub_Mpb70_R  MycoTub_Mpb70_P | CTCAATCCGCAAGTAAACC TCAGCAGTGACGAATTGG FAM-CTCAACAGCGGTCAGTACACGGT-BHQ1 | 133 | Lorente-Leal et al., (2019) | culture |
| *Pasteurella multocida*  *(2)* | kmt1 | Pastmulto_kmt1_F  Pastmulto_kmt1_R  Pastmulto_kmt1_P | AACCGGCAAATAACAATAAGCTG  TGAGTGGGCTTGTCGGTAG  FAM-AGTAATAAATAACGTCCAATCAGTTGCGCCGT-BHQ1 | 156 | This study | culture |
| *Salmonella* spp.  *(2)* | ttr | SalmoSp_ttr_F  SalmoSp_ttr_R  SalmoSp_ttr_P | CTCACCAGGAGATTACAACATGG AGCTCAGACCAAAAGTGACCATC FAM-CACCGACGGCGAGACCGACTTT-BHQ1 | 95 | Malorny et al., (2004) | culture |
| *Salmonella enterica subsp. enterica* serovar Enteritidis | SEN1383 | SalmoEnteri_ SEN1383_F  SalmoEnteri_ SEN1383_R  SalmoEnteri_ SEN1383_P | GACGCCAAAAAGCGAGACCT TAACTCTTCGGGTTTAACTCTCA FAM-CAAACTTACTCAGGAGATCGCCGCTG-BHQ1 | 165 | Adapted from Richmond et al., (2011) | culture |
| *Salmonella enterica subsp. enterica* serovar Typhimurium | LysR | SalmoTyphim_ LysR _F  SalmoTyphim_ LysR _R  SalmoTyphim_ LysR _P | CTACATTCCTTCCTGATATTGTG  TCCAGCATTATTTTGTTAGCGTG  FAM-TGATGAATTACATTATGGTCGGGCAGCCAG-BHQ1 | 206 |  | culture |
| Pathogenic *Yersinia enterocolitica* | ail | YersiEntero_ail_F  YersiEntero_ail_R  YersiEntero_ail_P | AAGTACCGTTATGAACTCGATGA CCCAGTAATCCATAAAGGCTAAC  FAM-TAACTGGGGAGTAATAGGTTCGTTTGCTTATAC-BHQ1 | 183 | Adapted from Sannö et al., (2014) | culture |
| *Yersinia* spp. | 1) Glna | YersiSp1_glna_F  YersiSp1_glna_R  YersiSp1_glna_P | TCCAGCACCAAATACGAAGGT CACATGGCAGAACGCAGATC  FAM-TTGCGCGGAATCAACTGGGGGAACC-BHQ1 | 110 | Adapted from Keeling et al., (2012) | synthetic oligonucleotide & culture |
|  | 2) ompF | YersiSp2_ompF_F  YersiSp2_ompF_R  YersiSp2_ompF_P | GTCTGGGCTTTGCTGGTCT AGTTGTCAGAGTTGGAGATTGAA FAM-TCACCACCGAACACTGGCAGCATGT-BHQ1 | 141 | Adapted from Hashemi et al., (2016) | synthetic oligonucleotide & culture |
|  | 3) ompF | YersiSp3_ompF_F  YersiSp3_ompF_R  YersiSp3_ompF_P | CTTGCAGTAGTAATCCCAGCAT TCAAGTTTGTTGCCGTCTTTGTT FAM-TAGATTTCTGCTGCATTAGCTGCGCCAG-BHQ1 | 83 | This study | synthetic oligonucleotide & culture |
| Control for inhibitor factors | | | | | | |
| Enhanced Green Fluorescent Protein | EGFP | EGFP_htrtPCR_F  EGFP_ htrtPCR _R  EGFP_ htrtPCR _P | CACTACCAGCAGAACACCC  CTTGTACAGCTCGTCCATGC  FAM-CCATCGGCGACGGCCCCGTG-BHQ1 | 174 | Hoffmann et al., (2006) | synthetic oligonucleotide |

**References**

Aaziz, R., Laroucau, K., Gobbo, F., Salvatore, D., Schnee, C., Terregino, C., Lupini, C., & Di Francesco, A. (2022). Occurrence of Chlamydiae in Corvids in Northeast Italy. *Animals*, *12*(10), Article 10. https://doi.org/10.3390/ani12101226

Bounaadja, L., Albert, D., Chénais, B., Hénault, S., Zygmunt, M. S., Poliak, S., & Garin-Bastuji, B. (2009). Real-time PCR for identification of Brucella spp.: A comparative study of IS711, bcsp31 and per target genes. *Veterinary Microbiology*, *137*(1–2), 156–164. https://doi.org/10.1016/j.vetmic.2008.12.023

Cerdà-Cuéllar, M., Moré, E., Ayats, T., Aguilera, M., Muñoz-González, S., Antilles, N., Ryan, P. G., & González-Solís, J. (2019). Do humans spread zoonotic enteric bacteria in Antarctica? *Science of The Total Environment*, *654*, 190–196. https://doi.org/10.1016/j.scitotenv.2018.10.272

Challier, S., Boyer, S., Abachin, E., & Berche, P. (2004). *Development of aserum-based taqman real-time PCR assay for diagnosis of invasive aspergillosis*. *42*(2), 844–846.

Ehricht, R., Slickers, P., Goellner, S., Hotzel, H., & Sachse, K. (2006). Optimized DNA microarray assay allows detection and genotyping of single PCR-amplifiable target copies. *Molecular and Cellular Probes*, *20*(1), 60–63. https://doi.org/10.1016/j.mcp.2005.09.003

Galvani, A. T., Christ, A. P. G., Padula, J. A., Barbosa, M. R. F., De Araújo, R. S., Sato, M. I. Z., & Razzolini, M. T. P. (2019). Real-time PCR detection of Toxoplasma gondii in surface water samples in São Paulo, Brazil. *Parasitology Research*, *118*(2), 631–640. https://doi.org/10.1007/s00436-018-6185-z

Hashemi, S., Mahzounieh, M., & Ghorbani, M. (2016). *Detection of Yersinia spp and Salmonella spp. In apparently healthy cats and dogs in Tehran, Iran*. *4*(16).

Hoffmann, B., Depner, K., Schirrmeier, H., & Beer, M. (2006). A universal heterologous internal control system for duplex real-time RT-PCR assays used in a detection system for pestiviruses. *Journal of Virological Methods*, *136*(1), 200–209. https://doi.org/10.1016/j.jviromet.2006.05.020

Karlsson, I., Edel-Hermann, V., Gautheron, N., Durling, M. B., Kolseth, A.-K., Steinberg, C., Persson, P., & Friberg, H. (2016). Genus-Specific Primers for Study of Fusarium Communities in Field Samples. *Applied and Environmental Microbiology*, *82*(2), 491–501. https://doi.org/10.1128/AEM.02748-15

Keeling, S. E., Johnston, C., Wallis, R., Brosnahan, C. L., Gudkovs, N., & McDonald, W. L. (2012). Development and validation of real-time PCR for the detection of Yersinia ruckeri. *Journal of Fish Diseases*, *35*(2), 119–125. https://doi.org/10.1111/j.1365-2761.2011.01327.x

Lorente-Leal, V., Liandris, E., Castellanos, E., Bezos, J., Domínguez, L., De Juan, L., & Romero, B. (2019). Validation of a Real-Time PCR for the Detection of Mycobacterium tuberculosis Complex Members in Bovine Tissue Samples. *Frontiers in Veterinary Science*, *6*, 61. https://doi.org/10.3389/fvets.2019.00061

Malorny, B., Paccassoni, E., Fach, P., Bunge, C., Martin, A., & Helmuth, R. (2004). Diagnostic Real-Time PCR for Detection of *Salmonella* in Food. *Applied and Environmental Microbiology*, *70*(12), 7046–7052. https://doi.org/10.1128/AEM.70.12.7046-7052.2004

Michelet, L., De Cruz, K., Karoui, C., Tambosco, J., Moyen, J.-L., Hénault, S., & Boschiroli, M. L. (2018). Second line molecular diagnosis for bovine tuberculosis to improve diagnostic schemes. *PLOS ONE*, *13*(11), e0207614. https://doi.org/10.1371/journal.pone.0207614

Michelet, L., Delannoy, S., Devillers, E., Umhang, G., Aspan, A., Juremalm, M., Chirico, J., van der Wal, F. J., Sprong, H., Boye Pihl, T. P., Klitgaard, K., BÃ¸dker, R., Fach, P., & Moutailler, S. (2014). High-throughput screening of tick-borne pathogens in Europe. *Frontiers in Cellular and Infection Microbiology*, *4*. https://doi.org/10.3389/fcimb.2014.00103

Pal, N., Bender, J. S., & Opriessnig, T. (2010). Rapid detection and differentiation of *Erysipelothrix* spp. By a novel multiplex real‐time PCR assay. *Journal of Applied Microbiology*, *108*(3), 1083–1093. https://doi.org/10.1111/j.1365-2672.2009.04560.x

Radomski, N., Roguet, A., Lucas, F. S., Veyrier, F. J., Cambau, E., Accrombessi, H., Moilleron, R., Behr, M. A., & Moulin, L. (2013). atpE gene as a new useful specific molecular target to quantify Mycobacterium in environmental samples. *BMC Microbiology*, *13*(1), 277. https://doi.org/10.1186/1471-2180-13-277

Richard, F.-J., Southern, I., Gigauri, M., Bellini, G., Rojas, O., & Runde, A. (2021). Warning on nine pollutants and their effects on avian communities. *Global Ecology and Conservation*, *32*, e01898. https://doi.org/10.1016/j.gecco.2021.e01898

Richmond, G. S., Khine, H., Zhou, T. T., Ryan, D. E., Brand, T., McBride, M. T., & Killeen, K. (2011). MassCode Liquid Arrays as a Tool for Multiplexed High-Throughput Genetic Profiling. *PLOS ONE*, *6*(4), e18967. https://doi.org/10.1371/journal.pone.0018967

Sannö, A., Aspán, A., Hestvik, G., & Jacobson, M. (2014). Presence of *Salmonella* spp., *Yersinia enterocolitica* , *Yersinia pseudotuberculosis* and *Escherichia coli* O157:H7 in wild boars. *Epidemiology and Infection*, *142*(12), 2542–2547. https://doi.org/10.1017/S0950268814000119

**Note on extraction control**: Finding a common target between phylogenetically distant hosts (birds, mammals) was challenging. We identified a PCR system targeting the B-actin that works on both avian and mammalian DNA. However, for cost and analytical reasons, we chose not to test all samples with B-actin. We used a Flex extraction automat for DNA extraction, which allows a standardised and repeatable experiment and an identical extraction quality for all samples from the same plate (fewer experimental errors than with manual extraction). 2) We randomly tested 10% of the DNA from each plate with B-actin PCR systems and no extraction failures were detected, arguing for correct extraction for all samples, 3) Most samples tested positive for at least one IA using Htrt PCR and 4) Inhibitory factors were assessed with the dedicated EGFP PCR system.

**Note on cross contamination:**  We worked in a laboratory with its own quality reference analysis protocols, as it is a national and international reference laboratory for 6 and 4 bacterial pathogens, respectively. To minimise cross-contamination, we worked in dedicated areas with separate equipment and protective clothing (including gloves and filter tips), carefully loading samples and PCR systems onto chips, and critically reviewing results. We used a forward approach within the laboratory, with a first room for extraction, then a room for PCR mix, a room for dilution of pre-amplification products and a final room for chip preparation. We also used internal controls to check for cross-contamination.

**Supporting Information – S2 | List of criteria for the design of primers/probes for Htrt PCR and reference genomes (GenBank) used for newly designed primers/probes for this study.**

| **Infectious agent** | **Target** | **GenBank accession number** |
| --- | --- | --- |
| *Aspergillus fumigatus* | 28S rRNA | MN625298.1, MN134347.1, JQ965824.1, MT625993.1, MN190286.1 + KM213866.1 (*Penicillium* spp.) |
| *Mucorales* | 18S rRNA | NG063234.1, MH931263.1, NG061009.1, MH864361.1, MG760354.1, AF113432.1, NG063355.1, AF157146.1, NG077400.1, MK300698.1, LC485157.1, MT649537.1, MK623262.1, MK849618.1, MN685272.1 |
| *Fusarium* spp. | TEF1 | ON037537.1, MK152502.1, XM_031186851.2, MN689178.1, ON932354.1, MN507111.1, OP715611.1, ON454030.1, ON292427.1, OL828606.1, OL828699.1 |
| *Campylobacter coli* | lpxA | CP019977.1, CP023545.1, CP038868.1, CP044165.1 |
| *Campylobacter jejuni* | mapA | AP025981.1, OX437060.1, OX437058.1, CP053659.1 |
| *Campylobacter lari* | lpxA | CP044161.1, AY598984.1, AY598983.1, CP053854.1, AY531475.1, AY531481.1, AY531485.1 |
| *Chlamydiaceae* | 23S rRNA | MH974825.1 |
| *Chlamydia psittaci* | enoA | CP025423.1, CP110211.1, CP047319.1, CP098512.1 |
| *Erysipelothrix amsterdamensis* | 5S rRNA | OW659477.1 |
| *Erysipelothrix rhusiopathiae* | 5S rRNA | CP029804.1, CP033601.1, CP005079.1, CP041995.1 + AB019248.1, LC528614.1 (*E. tonsillarum*) + OW659477.1 (*E. amsterdamensis*) |
| *Pasteurella multocida* | kmt1 | CP097610.1, CP037865.1, CP020347.1, LR134514.1, KY825088.1, LR134298.1, CP014157.1, |
| *Samonella enterica subsp. enterica* serovar Enteritidis | SEN1383 | CP041176.1, CP032851.1, CP050716.1, CP015524.1, CP007332.2 |
| *Samonella enterica subsp. enterica* serovar Typhimurium | LysR | CP123698.1, CP119867.1, CP117398.1, CP112994.1 + CP117301.1 (serovar Derby) + CP119481.1 (serovar Agona) + CP123663.1, CP119501.1 (serovar Newport) |
| Pathogenic *Yersinia enterocolitica* | ail | FR847859.1, HQ419068.1, KU672532.1, KT209984.1, KP288671.1, FN812734.1, JQ665437.1, JX972143.1, JX972144.1, KM253266.1, KM253257.1, MZ491081.1 |
| *Yersinia* spp. | 1) Glna | DQ400831.1, DQ400780.1 (*Y. entomophaga*) + MH156841.1, KP894095.1 (*Y. ruckeri*) |
|  | 2) ompF | CP096666.1, CP054049.1, AP024605.1, CP071944.1, CP009787.1, CP023964.1, CP022338.1, CP107097.1 |
|  | 3) ompF | HM142667.1, CP010029.1, CP011975.1, HM142653.1, CP009781.1, CP054043.1 |
| Enhanced Green Fluorescent Protein | EGFP | LC783902.1 |


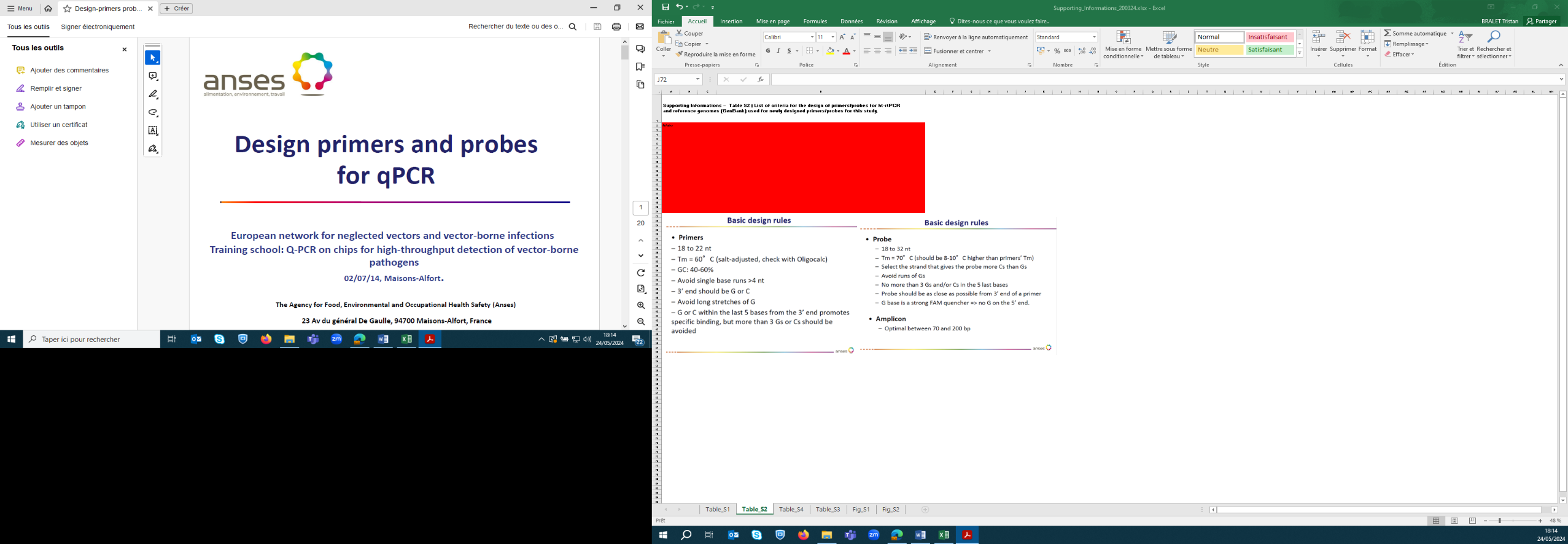

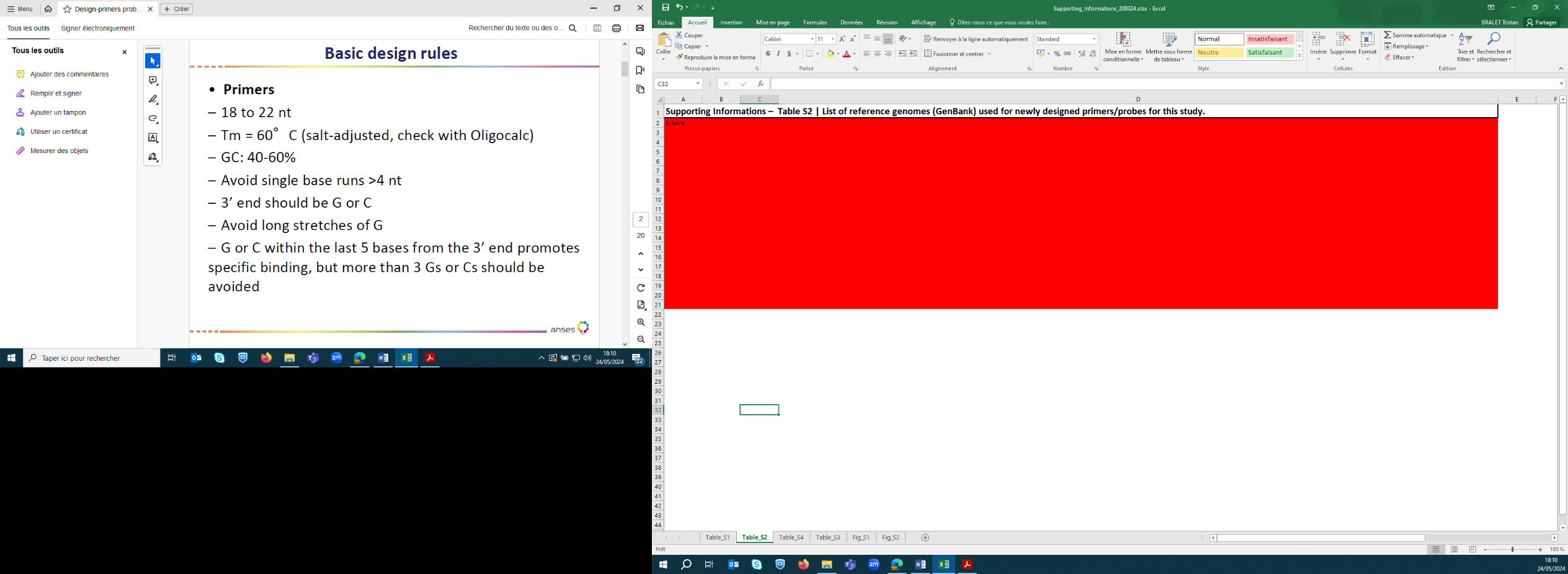

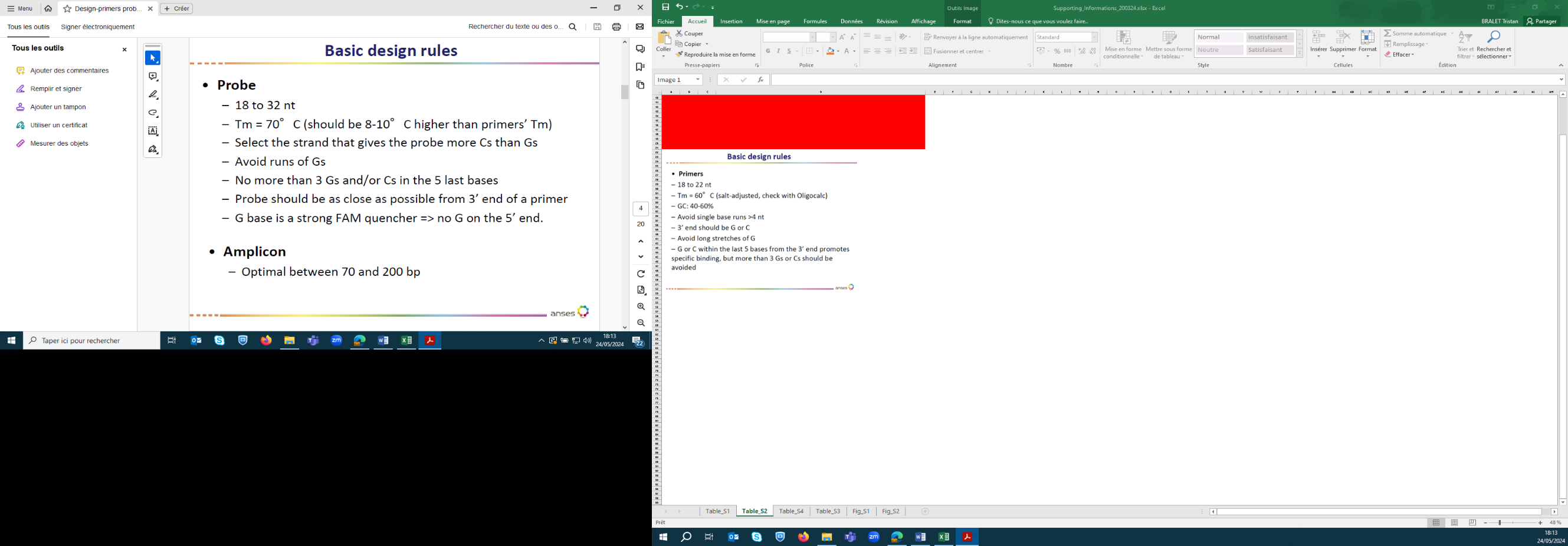


**Supporting Information – S3 | List of reference genomes used for newly designed primers/probes for this study and origin of positive controls used for validation**. np.: Information non provided.

| **Cultures** | | | |
| --- | --- | --- | --- |
| **Species** | **Positive controls** | **References** | **ID** |
| *Erysipelothrix amsterdamensis* | A18Y020d, GenBank : Q355811 | Zhong et al. 2024 | ADNctrl001 |
| *Pasteurella multocida* | Culture from field necropsies, identification by spectrometry (MALDI-TOF) | This study (Amsterdam Island) | ADNctrl002 |
|  | Culture from field necropsies, identification by spectrometry (MALDI-TOF) | This study (Amsterdam Island) | ADNctrl003 |
| *Salmonella enterica* serovar Typhimurium | 260 Holland | Sabine Delannoy (COLiPATH Unit & Genomics Platform IdentyPath, Laboratory for Food Safety, ANSES, 94700 Maisons-Alfort, France) | ADNctrl005 |
| *Campylobacter coli* | np. | David Albert (French Food Safety Agency, LCSV Unit - Central Veterinay Services Laboratory, Laboratory for Food Safety (LSAL), Maisons-Alfort F-94700, France) | ADNctrl006 |
| *Campylobacter lari* | np. |  | ADNctrl007 |
| *Campylobacter jejuni* | np. |  | ADNctrl008 |
| *Aspergillus fumigatus* | ATCC Af13073 | Bralet et. al, 2023 | ADNctrl009 |
| *Mucorales spp.* | *Lichteimia sp*. 2222870094 | Guillaume Désoubeaux (Parasitology and Mycology Department, Bretonneau Hospital, 37032, Tours, France Boulevard Tonnellé, University of Tours, 37032, Tours, France) | ADNctrl010 |
|  | *Rhizopus arrizhus* 2229170036 |  | ADNctrl011 |
|  | *Mucor circinelloides* 2229270036 |  | ADNctrl012 |
| *Fusarium spp.* | *Fusarium sp*. 2229370048 |  | ADNctrl013 |
| **Synthetic oligonucleotides** | | | |
| **Plasmid** | **Sequence/Reference** | | **ID** |
| Pathogenic *Yersinia enterocolitica* | AAGTACCGTTATGAACTCGATGATAACTGGGGAGTAATAGGTTCGTTTGCTTATACCCATCAGGGATACGATTTCTTCTATGGCAGTAATAAGTTTGGTCACGGTGATCTTGATTACTATTCAGTAACAATGGGGCCATCTTTCCGCATTAACGAATATGTTAGCCTTTATGGATTACTGGGTCCAGCACCAAATACGAAGGTGGTAACAAAGGCCATCGTCCCGCAGTAAAAGGCGGTTACTTCCCGGTTCCCCCAGTTGATTCCGCGCAAGATCTGCGTTCTGCCATGTGCTTGCAGTAGTAATCCCAGCATTGTTAGCAGCTGGCGCAGCTAATGCAGCAGAAATCTACAACAAAGACGGCAACAAACTTGA/This Study | | P1 |
| *Yersinia spp. (1) (Glna)* |  |  |  |
| *Yersinia spp. (3) ompF* |  |  |  |
| *Borrelia garinii (rpoB)* | AGGCCCGTCCGTTATTTTACGGGTGTGCCAAGCCCGGTCAAAACGCCTGAAAAGGTGAATATGGTGATTTTCCGGAGTCTTAAAAGGGCGATTTAGTTAGATGTGGTAGACCCGAAGCCGAGTGATCTATTTATGGCCAGGCTGAAGGGAGCAAATCAAGATGAAGCAATTGCTGTAAATATTTATTCAGCTAATGTTGCAAATCTTTTTGCTGGTGAGGGAGCTCAAGCTGCTCAGGCTGCACCTGTTCAAGAGGGTGCTCAGCTTACTCACAAAAGGCGTCTTAATGCTCTTGGACCAGGAGGACTTTCAAGAGATAGGGCAGGATTTGAAGTAAGAGATGTGCTGGCCGAACTTACCCACAAAAGGCGTCTTAATGCCCTTGGACCAGGGGGACTTTCAAGAGATAGAGCAGGATTTGAAGTAAGAGATGT/This Study | | P2 |
| *Borrelia azfelli (fla)* |  |  |  |
| *Borrelia burgdorferi ss (rpoB)* |  |  |  |
| *Borrelia spp. (23S rRNA)* |  |  |  |
| *Coxiella burnetii (Icd)* |  |  |  |
| Pathogenic *Leptospira spp. (LipL32)* | GGATCTGTGATCAACTATTACGGATATGTAAAGCCAGGACAAGCGCCGGACGGTTTAGTCGACGGAAGCAAAAAAGCATACTATCTCTATATTTGGATCCCAACTGTAATCGCCGAAATGGGAGTTCGTATGATTTCCCCAACGGCCACAATGGAACTGAGACACGGTCCATACTCCTACGGGAGGCAGCAGTTAAGAATCTTGCTCAATGGGGTGAAATGTACCTCCAGAAAAGCCACCTAGTATCGTGCGGCAATGTGCCACCTCGCCTCTTGGGAGAAAAAGAGGAAGAGACGCTGCC/This Study | | P3 |
| *Leptospira spp. (16S)* |  |  |  |
| *Toxoplasma gondii (B1)* |  |  |  |
| *Yersinia spp. (ompF)* | GTCTGGGCTTTGCTGGTCTGAAATTTGCTGAATTCGGTTCATTCGACTATGGCCGTAACTACGGCGTAATCTATGACGTTAACGCATGGACTGACATGCTGCCAGTGTTCGGTGGTGATTCAATCTCCAACTCTGACAACT/This Study | | P4 |
| *Yersinia spp. (rpoB)* | AAGTACCGTTATGAACTCGATGATAACTGGGGAGTAATAGGTTCGTTTGCTTATACCCATCAGGGATACGATTTCTTCTATGGCAGTAATAAGTTTGGTCACGGTGATCTTGATTACTATTCAGTAACAATGGGGCCATCTTTCCGCATTAACGAATATGTTAGCCTTTATGGATTACTGGGTCCAGCACCAAATACGAAGGTGGTAACAAAGGCCATCGTCCCGCAGTAAAAGGCGGTTACTTCCCGGTTCCCCCAGTTGATTCCGCGCAAGATCTGCGTTCTGCCATGTGCTTGCAGTAGTAATCCCAGCATTGTTAGCAGCTGGCGCAGCTAATGCAGCAGAAATCTACAACAAAGACGGCAACAAACTTGA/This Study | | P5 |
| *Salmonella spp. (invA)* |  |  |  |
| *Leptospira spp. (rrs)* |  |  |  |
| EGFP | CACTACCAGCAGAACACCCCCATCGGCGACGGCCCCGTGCTGCTGCCCGACAACCACTACCTGAGCACCCAGTCCGCCCTGAGCAAAGACCCCAACGAGAAGCGCGATCACATGGTCCTGCTGGAGTTCGTGACCGCCGCCGGGATCACTCTCGGCATGGACGAGCTGTACAAG | | pEGFP |
| *Chlamydiaceae* | See Aaziz et al. 2015 | | pCH23S |
| **DNA extracts** | | | |
| **Species** | **Positive controls** | **References** | **ID** |
| *Salmonella enterica* serovar Enteritidis | np. | Sabrina Cadel-Six (Salmonella and Listeria Unit (SEL), ANSES, Laboratory for Food Safety, Maisons-Alfort, France) | ADNctrl014 |
| *Erysipelothrix rhusiopathiae* | *E. rhusiopathiae* ERY 0220 clade intermédiaire (swine) | Taya Forde (School of Biodiversity, One Health & Veterinary Medicine, University of Glasgow, Glasgow, G12 8QQ, UK) | ADNctrl015 |
|  | *E. rhusiopathiae* ERY 0026 clade 3 (fish) |  | ADNctrl016 |
|  | *E. rhusiopathiae* ERY 0001 clade 3 (swine) |  | ADNctrl017 |
|  | *E. rhusiopathiae* ERY 0025 clade 1 (fish) |  | ADNctrl018 |
|  | *E. rhusiopathiae* ERY 0223 clade 3 (fish) |  | ADNctrl019 |
|  | *E. rhusiopathiae* ERY 0082 clade 2 (swine) |  | ADNctrl020 |
|  | *E. rhusiopathiae* ERY 0038 clade 1 (sheep) |  | ADNctrl021 |
|  | *E. rhusiopathiae* ERY 0021 clade 2 (fish) |  | ADNctrl022 |
| *Erysipelothrix tonsillarum* | *E. tonsillarum* ERY 0041 (fish) |  | ADNctrl023 |
| *Chlamydia psittaci* | C. psittaci Loth | University Paris-Est, Anses, Animal Health Laboratory, Bacterial Zoonoses Unit, Maisons-Alfort, France | Loth |
| *Mycobacterium bovis* complex | M. bovis D-11-01143 (11Z2135) | Lorraine Michelet (National Reference Laboratory for Animal Tuberculosis, Animal Health Laboratory, Paris-Est University/ANSES, Maisons-Alfort, France) | ADNctrl024 |
| *Mycobacterium avium* complex | M. avium D-21-02197 (21Z003583) |  | ADNctrl025 |
| *Brucella spp.* | *Brucella ovis* 63/290 | National Reference Laboratory for Animal Brucellosis, Animal Health Laboratory, Paris-Est University/ANSES, Maisons-Alfort, France | ADNctrl026 |
| *Toxoplasma gondii* | *T. gondii* RH (type 1) | Aurélien Mercier (Centre National de Référence Toxoplasmose, CHU Limoges, 2 Martin Luther King Street, 87042 Limoges, France) | ADNctrl027 |
|  | *T. gondii* ME49 (type 2) |  | ADNctrl028 |
|  | *T. gondii* NED (type 3) |  | ADNctrl029 |
| *Leptospira spp.* | *Leptospira interrogans icterohemorragiae strain copenhageni winjberg* | Mathieu Picardeau (Institut Pasteur, Université Paris Cité, CNRS UMR 6047, Biology of Spirochetes Unit, Paris, France) | ADNctrl032 |
|  | *Leptospira biflexa serovar Patoc strain Patoc 1* |  | ADNctrl033 |
|  | *Leptospira noguchii serovar Panama strain CZ214K* |  | ADNctrl034 |
|  | *Leptospira kirschneri serovar Grippotyphosa strain Moskva V* |  | ADNctrl035 |
|  | *Leptospira borgpetersenii serovar Ballum strain Castellon 3* |  | ADNctrl036 |
|  | *Leptospira interrogans serovar Australis strain Ballico* |  | ADNctrl037 |
|  | *Leptospira santarosai serogroup Celledoni strain 2011/01963* |  | ADNctrl038 |
|  | *Leptospira weilii serovar Sarmin strain Sarmin* |  | ADNctrl039 |
|  | *Leptospira noguchii serovar Louisiana strain louisiana* |  | ADNctrl040 |
|  | *Leptospira kischneri serovar cynopteri strain 3522C* |  | ADNctrl041 |
| *Yersinia entomophaga* | IP36721 | Javier Pizarro-Cerda (Institut Pasteur, Université Paris Cité, Yersinia National Reference Laboratory, WHO Collaborating Research & Reference Centre for Plague FRA-140, Paris, France) | ADNctrl042 |
| *Yersinia pseudotuberculosis* | IP32953 |  | ADNctrl043 |
| *Yersinia intermedia* | IP 35753 |  | ADNctrl044 |
| *Yersinia enterocolitica* | IP 33698 |  | ADNctrl045 |

**Supporting Information – S4 | A. Simultaneously obtained prevalences by Htrt PCR (after validation step) for 25 infectious agents on samples from systematic sampling (apparently healthy individuals).** Zero prevalences are not shown.

**B. Qualitative results of Htrt PCR performed on necropsy material from passive surveillance (opportunistically found carrions)**. Only samples with positive PCR results are reported.

**Supporting Information – S5 | BioMark™ dynamyc array system specificity test (48.48 chip)**. Only reactions with Ct < 30 are considered positives and are shown.
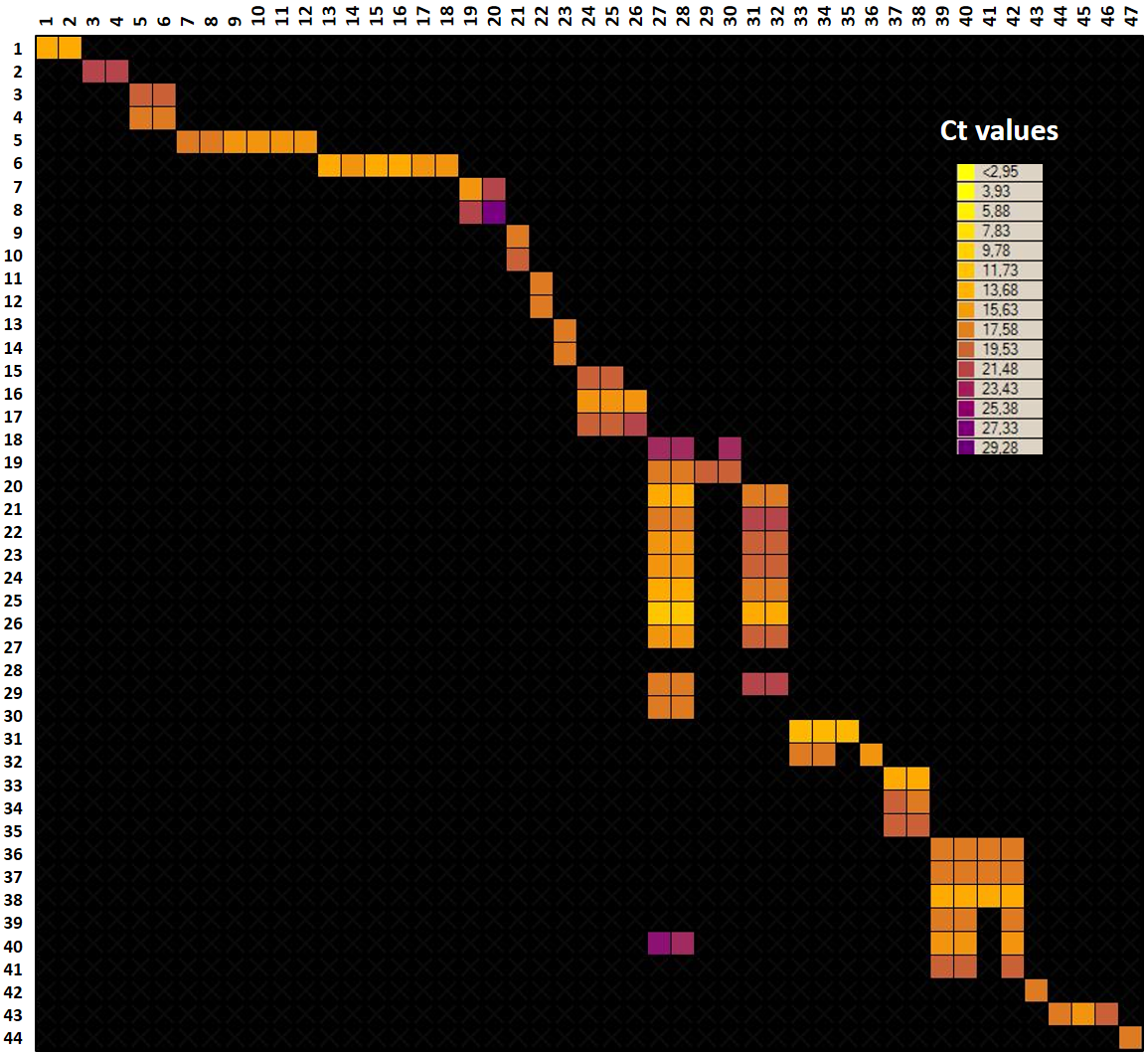

**Supporting Information – S6 | Primers used to confirm the presence of infectious agent DNA in samples using qPCR or endpoint PCR followed by gene sequencing**. For each IA, validation steps were only performed on a representative batch of Htrt PCR positive samples (see dedicated column) and results were extrapolated to all samples. np. : Information not provided

| **Species** | **Method** | **Sequence** | **Target** | **Reference** | **Comments** | **Number of Htrt PCR positive samples** | **Number of tested samples (validation step)** | **Number of successful sequencing** | **Number of positive samples (validation step)** |
| --- | --- | --- | --- | --- | --- | --- | --- | --- | --- |
| *Mucorales* | qPCR | np. | np. | MycoGENIE® Aspergillus- Mucorales spp. Real-Time PCR Kit | Commercial kit : confirmation by Parasitology and Mycology Department, Bretonneau Hospital, 37032, Tours, France | 4 | 4 | 2 | 2 |
| *P. multocida* | qPCR | Pm_Jaeger_F ACGGCGCAACTGATTGGACG Pm_Jaeger_R GGCCATAAGAAACGTAACTCAACA Pm_Jaeger_S TCAGCTTATTGTTATTTGCCGGT | kmt1 | Jaeger et al., 2020 | - | 85 | 25 | - | 25 |
|  | Gene sequencing | Pastmulto_kmt1_F AACCGGCAAATAACAATAAGCTG Pastmulto_kmt1_R TGAGTGGGCTTGTCGGTAG | kmt1 | This study | - |  | 6 | 3 | 3 |
| *E. rhusiopathiae* | Gene sequencing | ErysiRhu_5S_F ATATGTTTTAGGTTCAGTCGTCC ErysiRhu_5S_R TGGACAAAGTCTAACGAAAGGTT | 5S | This study | - | 10 | 5 | 2 | 2 |
| *E.amsterdamensis* complex | Gene sequencing | MO101 AGATGCCATAGAAACTGGTA M0102 CTGTATCCGCCATAACTA | 16S | Makino et al. 1994 | - | 52 | 42 | 0 | - |
|  | Gene sequencing | ErysiAms_5S_F TCGTCAATTTCTAATCCCTTAGC ErysiAms_5S_R GTCTAAAGAAAGGGTGCCTCATT | 5S | This study | - |  | 20 | 0 | - |
|  | Gene sequencing | Erysiseq_5S_F GTCATACCGATTCTCAATCCAG Erysiseq_5S_R CATACTCAGTAACTGAGACAATG | 5S | This study | - |  | 19 | 1 | 1 (*E. enhydrae*) |
| *C. lari* | end-point PCR | CLF TAGAGAGATAGCAAAAGAGA CLR TACACATAATAATCCCACCC | glyA | Wang et al. 2002 | Confirmation by ANSES, Ploufragan-Plouzané Laboratory, Unit of Hygiene and Quality of Poultry and Pork Products, BP53, 22440 Ploufragan, France | 68 | 20 | - | 20 |
| *C. jejuni* | end-point PCR | CJF ACTTCTTTATTGCTTGCTGC  CJR GCCACAACAAGTAAAGAAGC | hipO |  |  | 5 | 5 | - | 0 |
|  | Gene sequencing | CampyLari_mapA_F GAGTAAAATTCATCCAAGTGCTG CampyLari_mapA_R GCTCCTACAAAAGAATAAGCTTC | mapA | This study | - |  | 5 | 2 | 2 |
| *Yersinia spp.* | qPCR | Yersi_Yang_F GGTGCTTCTCTGATTCCATTC TTG,  Yersi_Yang_R CGCCTGACGTTGCATGTTC  Yersi_Yang_S AACACGATGACGCCAACCGTGC | rpoB | Yang et al., 2016 | - | 64 | 32 | - | 32 |
| *S. enterica* serovar Enteritidis | qPCR | SalmoEnteri_bug_F TCGTACCTGCTGATGCTGGG SalmoEnteri_bug_R AGGATGAAGACGGGTAATGTCC SalmoEnteri_bug_P TATGCGCTGGTTCCGTTCCGTTTTCTGG | np. | Bugarel et al., 2017 | - | 5 | 5 | - | 5 |
|  | Gene sequencing | SalmoEnteri_ SEN1383_F GACGCCAAAAAGCGAGACCT SalmoEnteri_ SEN1383_R TAACTCTTCGGGTTTAACTCTCA | SEN1383 | This study | - |  | 5 | 1 | 0 |
| *S. enterica* serovar Typhimurium | qPCR | SalmoTyphi_bug_F AGATATTCCGTAGCAATTGAGTTG SalmoTyphi_bug_R AATAGCTAAAAATGACTGGGACTC SalmoTyphi_bug_P TGTGTTCAAGCAATGGTGAACAAACATAATCCC | np. | Bugarel et al., 2017 | - | 14 | 14 |  | 10 |
|  | Gene sequencing | SalmoTyphim_ LysR _F CTACATTCCTTCCTGATATTGTG SalmoTyphim_ LysR _R TCCAGCATTATTTTGTTAGCGTG | LysR | This study | - |  | 9 | 5 | 0 |
| *S. enterica* serovar Newport | qPCR | SalmoNewp_bug_F AATGGCTGGTAGCCTGTTCG SalmoNewp_bug_R AGGGAAAGCAAGGAACAGTAG SalmoNewp_bug_P TCATGCTATGCACTGGGAACAATTTCTGGC | np. | Bugarel et al., 2017 | - | - | 10 | - | 14 |
| *Salmonella spp.* | qPCR | Salmosp_Liang_F TTCCGCAACACATAGCCAAGC Salmosp_Liang_R AATCCAACAATCCATCAGCAAGG Salmosp_Liang_S TTTCTCCCCCTCTTCATGCGTTAC | invA | Liang et al., 2022 | - | 16 | 12 | - | 12 |
|  | Gene sequencing | Salmo_16s_F CCGTCWATTCMTTTGAGTTT Salmo_16S_R CTACGGGAGGCAGCAGT | 16S | Lane et al., 1991 | - |  | 14 | 7 | 7 |
| *Chlamydiaceae* | qPCR | Chlam_CH23S_F GAAACCAGTAGCTTATAAGCGGT Chlam_CH23S_R CTCGCCGTTTAACTTAACTCC Chlam_CH23S_P CTGGCTCATCATGCAAAAGGCACGC | 23S | Ehricht et al., 2006 | - | 125 | 22 | - | 22 |
| *C. psittaci* | qPCR | enoA_CpsF43 ATTCGCCCTATAGGTGCACAT enoA_CpsR162 5′-GCCTTCATCTCCAACTCCTGTAG enoA_CpsP79 GTGCGTATGGGTGCTGATGTTT | enoA | Aaziz et al., 2022 | - | 0 | 22 | - | 0 |
| *C. abortus* | qPCR | enoA_CabF13 AACAACGGCCTGCAATTTCAAG enoA_CabR124 TGAGAAGGTTTTTCAATGTATGGAAC enoA_CabP93 GGCACCCATACGTACAGCTTCTTG | np. |  | - | 0 | 22 | - | 0 |
| *Leptospira spp.* | qPCR | Leptospp_Mohd_F ACTGAGACACGGTCCATACT Leptospp_Mohd_R TAGTTAGCYGGTGCTTTAGGYA Leptospp_Mohd_S ACGGGAGGCAGCAGTTAAGAATCTTGC | rrs | Mohd Ali et al., 2018 | - | 12 | 12 | - | 11 |
|  | nested PCR and gene sequencing | Lepto_Merien_A GGCGGCGCGTCTTAAACATG Lepto_Merien_B TTCCCCCCATTGAGCAAGATT Lepto_Merien_C CAAGTCAAGCGGAGTAGCAA Lepto_Merien_D CTTAACCTGCTGCCTCCCGTA | 16S | Merien et al., 1992 | - |  | 5 | 3 | 3 |
| *Mycobacterium spp.* | Gene sequencing | Tbll ACCAACGATGGTGTGTCCAT  Tb12 CTTGTCGAACCGCATACCCT | hsp65 | Telenti et al. 1992 | Confirmation by National Reference Laboratory for Animal Tuberculosis, Animal Health Laboratory, Paris-Est University/ANSES, Maisons-Alfort, France | 166 | 20 | 19 | 19 |
| *Brucella spp.* | qPCR | IS421(Fd) CGCTCGCGCGGTGGAT IS511(Rv) CTTGAAGCTTGCGGACAGTCACC | IS711 | Confirmation by National Reference Laboratory for Animal Brucellosis, Animal Health Laboratory, Paris-Est University/ANSES, Maisons-Alfort, France | | 9 | 9 | - | 0 |

**Supporting Information – S7 | Validation of positive Htrt PCR results by single gene sequencing (Sanger sequencing) and best similarity with deposited sequences**. Only results with more than 90% similarity are shown. C: cloacal swab, O: oral swab; R: rectal swab; ++: More than one species with the same percentage of similarity.

| ID | Species | Sample type | targeted gene | *Identification* | Similarity (%) | Reference |
| --- | --- | --- | --- | --- | --- | --- |
| TB1-26 | king penguin | C | 16S | *Chlamydiifrater* spp. | 98.05 | LN810463  (common murre) |
| TB3-23 | macaroni penguin | C | 16S | *Chlamydiifrater* spp. | 100.00 | LN810463  (common murre) |
| TB20-20 | brown rat | R | 16S | *Chlamydia* spp. | 99.29 | KT012684  (ringed python) |
|  |  |  |  | *Chlamydia muridarum* | 99.15 | ++ |
| TB20-68 | subantarctic fur seal | R | 16S | *Chlamydia felis* | 99.66 | ++ |
| TB21-42 | Kerguelen shag | liver | ail | *Campylobacter jejuni* | 100.00 | ++ |
| TB21-42 | Kerguelen shag | bone marrow | ail | *Campylobacter jejuni* | 100.00 | ++ |
| TB2-45 | brown skua | C | 5S | *Erysipelothrix enhydrae* | 100.00 | NZ_CP125807  (Southern sea otter) |
| TB20-31 | subantarctic fur seal | O | 5S | *Erysipelothrix rhusiopathiae* | 100.00 | ++ |
| TB20-62 | subantarctic fur seal | R | 5S | *Erysipelothrix rhusiopathiae* | 100.00 | ++ |
| TB19-72 | brown rat | O | kmt1 | *Pasteurella multocida* | 100.00 | ++ |
| TB19-88 | brown rat | O | kmt1 | *Pasteurella multocida* | 100.00 | ++ |
| TB19-90 | brown rat | O | kmt1 | *Pasteurella multocida* | 100.00 | ++ |
| TB19-52 | house mouse | O | 16S | *Leptospira* spp. | 99.47 | KP822788  (bats) |
|  |  |  |  | *Leptospira interrogans* | 98.95 | ++ |
| TB2-76 | gentoo penguin | C | 16S | *Leptospira interrogans* | 94.23 | DQ522190  (environment) |
| TB20-70 | subantarctic fur seal | R | 16S | *Salmonella enterica subsp enterica* | 100.00 | ++ |
| TB20-67 | subantarctic fur seal | R | SEN1383 | *Salmonella enterica subsp enterica* | 100.00 | ++ |
| TB20-29 | brown rat | C | LysR | *Salmonella enterica subsp enterica* | 100.00 | ++ |
| TB20-38 | subantarctic fur seal | O | LysR | *Salmonella enterica subsp enterica* | 100.00 | ++ |
| TB20-61 | subantarctic fur seal | R | LysR | *Salmonella enterica subsp enterica* | 100.00 | ++ |
| TB20-63 | subantarctic fur seal | R | LysR | *Salmonella enterica subsp enterica* | 100.00 | ++ |
| TB20-64 | subantarctic fur seal | R | LysR | *Salmonella enterica subsp enterica* | 100.00 | ++ |
| TB1-39 | king penguin | C | hsp65 | *Mycobacterium lentiflavum* | 98.00 | ++ |
| TB2-56 | gentoo penguin | C | hsp65 | *Mycobacterium* spp. | 94.00 | ++ |
| TB2-73 | gentoo penguin | C | hsp65 | *Mycobacterium helvum* | 94.00 | ++ |
| TB2-88 | macaroni penguin | C | hsp65 | *Mycobacterium* spp. | 96.00 | ++ |
| TB3-02 | macaroni penguin | C | hsp65 | *Mycobacterium gordonae* | 93.00 | ++ |
| TB19-86 | brown rat | O | hsp65 | *Mycobacterium alsense* | 96.00 | ++ |
| TB20-13 | brown rat | R | hsp65 | *Mycobacterium genavense* | 96.00 | ++ |
| TB20-25 | brown rat | R | hsp65 | *Mycobacterium gordonae* | 96.00 | ++ |
| TB20-30 | brown rat | R | hsp65 | *Mycobacterium paraense* | 96.00 | ++ |
| TB20-36 | subantarctic fur seal | O | hsp65 | *Mycobacterium triviale* | 93.00 | ++ |

**Supporting Information – S8 | Epidemiological map of French Southern Territories** **with prevalences of three enteric IA obtained by Htrt PCR in avian mesopredators (top left) and apex avian predators and scavengers (top right) in Possession Island (Crozet Archipelago) and mammals from Kerguelen Archipelago (bottom left) and Amsterdam Island (bottom right).** Three IA of particular interest are represented (*Campylobacter lari* (yellow), *Salmonella spp*. (dark purple) and *Yersinia spp.* (pink)). Map were obtained on R with the ggmap and leaflet packages using the connectors ©Leaflet and ©Stadia Maps, Inc. Herein on ©OpenStreetMap (<https://tile.openstreetmap.org/>)


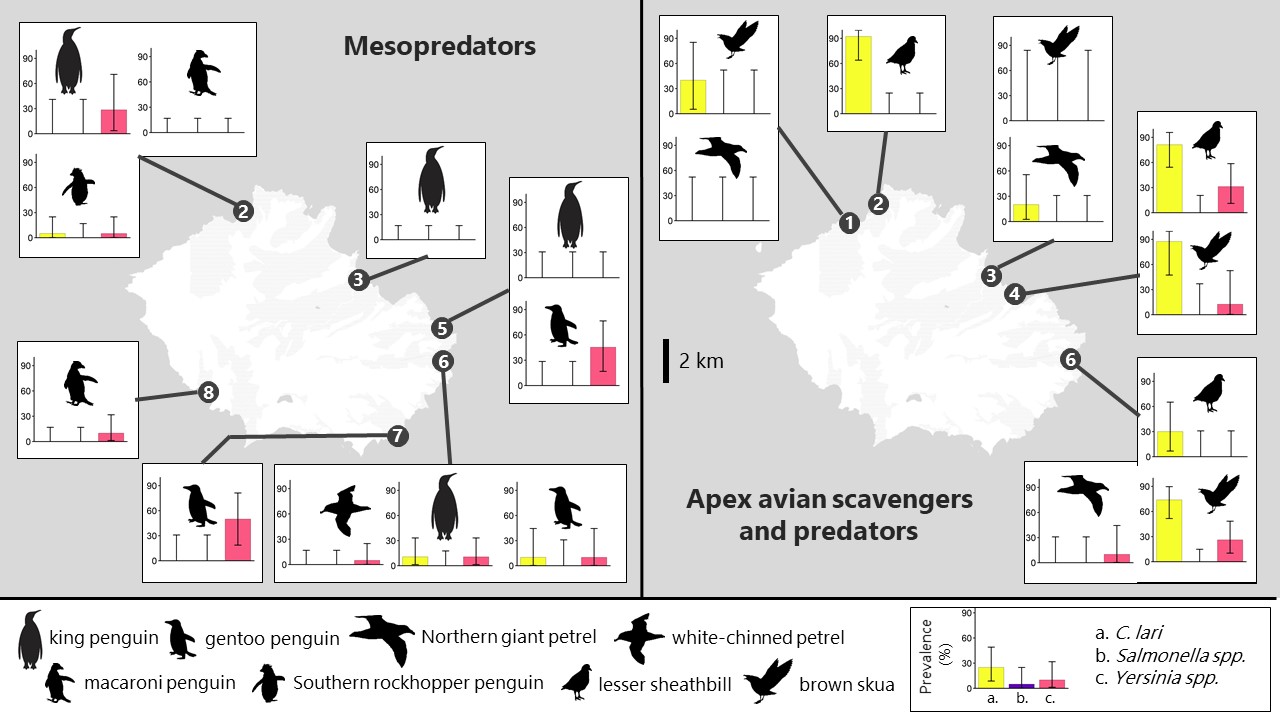


Samplig sites : 1: Pointe basse, 2: Jardin japonais, 3: Baie américaine, 4: Petite manchotière, 5: Crique de la chaloupe, 6: Baie du marin, 7: Crique de Noël, 8: Val austère.


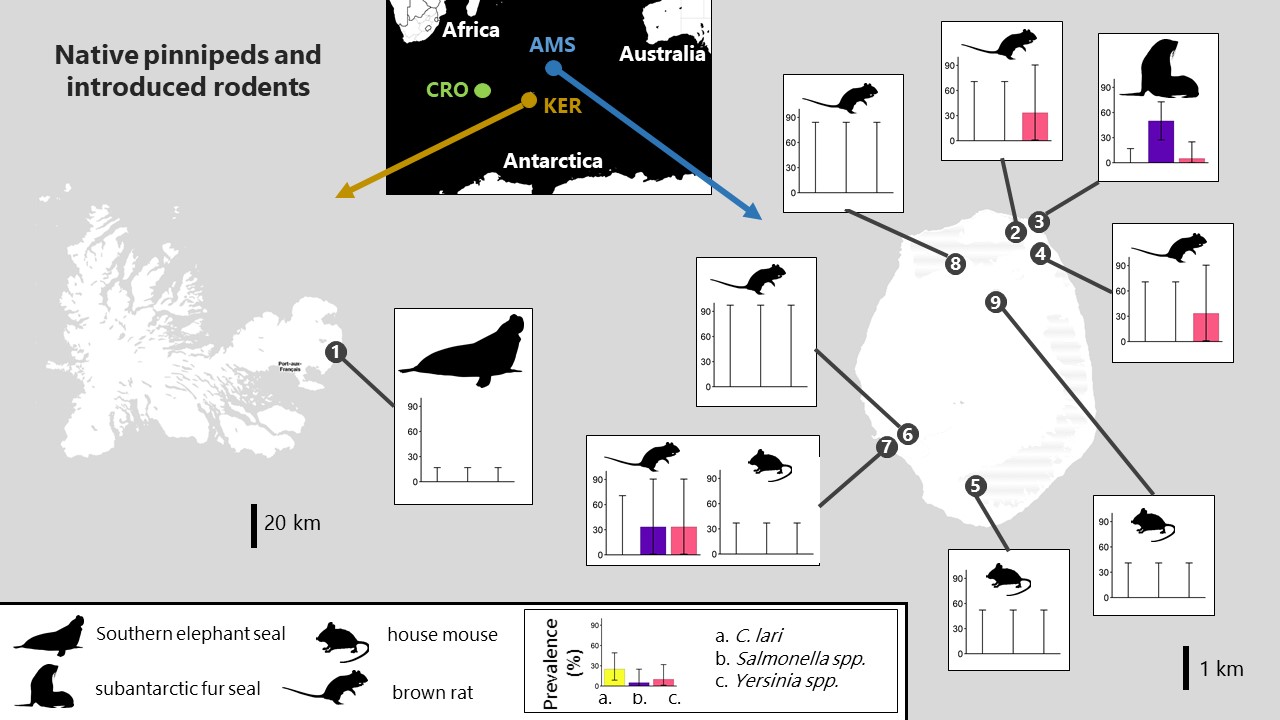


Sampling sites : 1: Estacade; 2 & 3: Scientific station Martin de Viviès; 4: Cratère Dumas; 5: Del Cano; 6 & 7: Entrecasteaux cliff; 8: Antonelli; 9: Plateau des tourbières

**Supporting Information – S9 | A. Generalised linear models parameters and summary (Figure 4) for comparisons of specific richness and Shannon index.**

| Linear mixed model fit by REML. t-tests use Satterthwaite's method ['lmerModLmerTest'] |
| --- |
| **Formula: as.numeric(Specific_Richness) ~ Species + (1 \| Site)** |
| Data: d |
|  |
| REML criterion at convergence: 762.6 |
|  |
| Scaled residuals: |
| Min 1Q Median 3Q Max |
| -2.6962 -0.5829 -0.1550 0.6050 2.6369 |
|  |
| Random effects: |
| Groups Name Variance Std.Dev. |
| Site (Intercept) 0.0254 0.1594 |
| Residual 0.9225 0.9605 |
| Number of obs: 274, groups: Site, 10 |
|  |
| Fixed effects: |
| Estimate Std. Error df t value Pr(>\|t\|) |
| (Intercept) 1.46975 0.20742 42.90457 7.086 9.78e-09 *** |
| Speciesgentoo_penguin -0.07094 0.27279 79.76304 -0.260 0.79548 |
| Speciesking_penguin -0.20080 0.24102 135.55663 -0.833 0.40624 |
| Specieslesser_sheathbill 1.01144 0.23782 241.31994 4.253 3.02e-05 *** |
| Speciesmacaroni_penguin -0.43863 0.27403 26.44068 -1.601 0.12134 |
| Speciesnorthern_giant_petrel -0.87122 0.27507 227.72449 -3.167 0.00175 ** |
| Speciessouthern_giant_petrel -0.38210 0.52651 246.76262 -0.726 0.46869 |
| Speciessouthern_rockhopper_penguin -0.94427 0.31934 48.59881 -2.957 0.00478 ** |
| Specieswhite_chinned_petrel -0.71975 0.33845 9.26517 -2.127 0.06150 . |
| --- |
| Signif. codes: 0 ‘***’ 0.001 ‘**’ 0.01 ‘*’ 0.05 ‘.’ 0.1 ‘ ’ 1 |
|  |
| Correlation of Fixed Effects: |
| (Intr) Spcsg_ Spcsk_ Spcsl_ Spcsm_ Spcsn__ Spcssthrn_g_ Spcssthrn_r_ |
| Spcsgnt_png -0.713 |
| Spcskng_png -0.788 0.617 |
| Spcslssr_sh -0.763 0.583 0.661 |
| Spcsmcrn_pn -0.741 0.536 0.612 0.613 |
| Spcsnrthr__ -0.673 0.512 0.604 0.559 0.508 |
| Spcssthrn_g_ -0.358 0.271 0.335 0.294 0.271 0.290 |
| Spcssthrn_r_ -0.627 0.458 0.534 0.546 0.574 0.435 0.232 |
| Spcswht_ch_ -0.613 0.437 0.483 0.467 0.454 0.412 0.220 0.384 |

| Linear mixed model fit by REML. t-tests use Satterthwaite's method ['lmerModLmerTest'] |
| --- |
| **Formula: as.numeric(Shannon_Index) ~ Species + (1 \| Site)** |
| Data: d |
|  |
| REML criterion at convergence: 289.9 |
|  |
| Scaled residuals: |
| Min 1Q Median 3Q Max |
| -2.1665 -0.6728 -0.2235 0.7933 2.5723 |
|  |
| Random effects: |
| Groups Name Variance Std.Dev. |
| Site (Intercept) 0.003103 0.0557 |
| Residual 0.155345 0.3941 |
| Number of obs: 274, groups: Site, 10 |
|  |
| Fixed effects: |
| Estimate Std. Error df t value Pr(>\|t\|) |
| (Intercept) 0.39319 0.08365 49.90247 4.701 2.08e-05 *** |
| Speciesgentoo_penguin -0.03131 0.11057 86.36618 -0.283 0.77772 |
| Speciesking_penguin -0.09226 0.09806 135.02402 -0.941 0.34849 |
| Specieslesser_sheathbill 0.42110 0.09724 241.78922 4.331 2.18e-05 *** |
| Speciesmacaroni_penguin -0.16913 0.10986 27.79463 -1.539 0.13500 |
| Speciesnorthern_giant_petrel -0.27403 0.11239 224.75806 -2.438 0.01553 * |
| Speciessouthern_giant_petrel -0.19639 0.21532 243.86599 -0.912 0.36264 |
| Speciessouthern_rockhopper_penguin -0.36486 0.12878 43.63607 -2.833 0.00695 ** |
| Specieswhite_chinned_petrel -0.35853 0.13367 11.33194 -2.682 0.02084 * |
| --- |
| Signif. codes: 0 ‘***’ 0.001 ‘**’ 0.01 ‘*’ 0.05 ‘.’ 0.1 ‘ ’ 1 |
|  |
| Correlation of Fixed Effects: |
| (Intr) Spcsg_ Spcsk_ Spcsl_ Spcsm_ Spcsn__ Spcssthrn_g_ Spcssthrn_r_ |
| Spcsgnt_png -0.721 |
| Spcskng_png -0.798 0.617 |
| Spcslssr_sh -0.775 0.589 0.665 |
| Spcsmcrn_pn -0.752 0.546 0.620 0.619 |
| Spcsnrthr__ -0.682 0.516 0.603 0.564 0.518 |
| Spcssthrn_g_ -0.362 0.272 0.330 0.296 0.275 0.285 |
| Spcssthrn_r_ -0.635 0.464 0.537 0.545 0.565 0.442 0.235 |
| Spcswht_ch_ -0.626 0.451 0.499 0.485 0.470 0.427 0.226 0.397 |

**
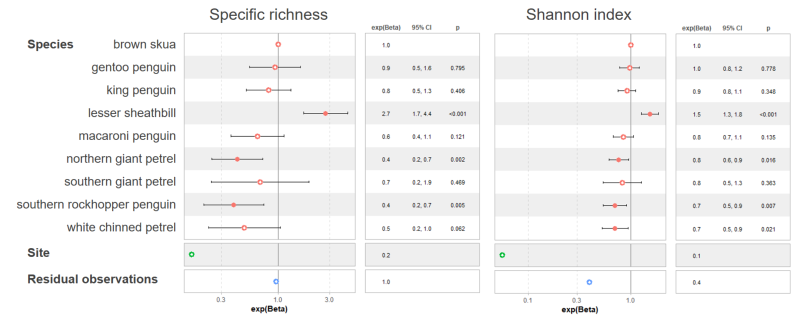
**

**B. Post hoc analyses: Pairwise comparison of specific richness (a.) and Shannon index (b.) of IA between species using non-parametric Kruskal-Wallis and then Wilcoxon tests.** Departure from normality using the Shapiro-Wilk test prevented the use of ANOVA methods.

**a.)**

> **kruskal.test(specific_richness ~ species, data = data)**

Kruskal-Wallis rank sum test

data: RS by espece

Kruskal-Wallis chi-squared = 78.673, df = 8, **p-value = 9.039e-14**

**> pairwise.wilcox.test(data$ specific_richness, data$species,+p.adjust.method = "BH")**

Pairwise comparisons using Wilcoxon rank sum test with continuity correction

data: data$RS and data$espece

brown_skua gentoo king sheathbill macaroni northern_giant southern_giant southern_rockhopper

gentoo_penguin 0.6788 - - - - - - -

king_penguin 0.3546 0.6788 - - - - - -

lesser_sheathbill **0.0020 0.0014 1.6e-06** - - - - -

macaroni_penguin 0.1344 0.3395 0.4639 **1.1e-06** - - - -

northern_giant_petrel **0.0020 0.0063 0.0045 2.1e-07** 0.0366 - - -

southern_giant_petrel 0.4453 0.6788 0.7247 **0.0465** 0.9899 0.3335 - -

southern_rockhopper_penguin **0.0010 0.0047 0.0029 2.9e-07 0.0234** 0.9899 0.2513 -

white_chinned_petrel **0.0115** 0.0840 0.0840 **1.6e-06** 0.3335 0.2489 0.6314 0.1599

P value adjustment method: BH

**b.)**

> kruskal.test(shannon ~ species, data = data)

Kruskal-Wallis rank sum test

data: shannon by espece

Kruskal-Wallis chi-squared = 80.273, df = 8, **p-value = 4.309e-14**

>

**> pairwise.wilcox.test(data$shannon, data$species,+ p.adjust.method = "BH")**

Pairwise comparisons using Wilcoxon rank sum test with continuity correction

data: data$shannon and data$species

brown_skua gentoo king sheathbill macaroni northern_giant southern_giant southern_rockhopper

gentoo_penguin 0.6834 - - - - - - -

king_penguin 0.3650 0.6365 - - - - - -

lesser_sheathbill **0.0024** **0.0016 1.1e-06** - - - - -

macaroni_penguin 0.1386 0.3682 0.5714 **1.1e-06** - - - -

northern_giant_petrel **0.0152** 0.0638 0.0830 **1.1e-06** 0.2531 - - -

southern_giant_petrel 0.4110 0.5900 0.6652 0.0523 0.8177 0.6899 - -

southern_rockhopper_penguin **0.0011** **0.0068** **0.0074** **1.1e-06 0.0181** 0.1251 0.0638 -

white_chinned_petrel **0.0035 0.0181 0.0205 1.1e-06**  0.0589 0.3682 0.3380 0.4247

P value adjustment method: BH

**Supporting Information - S10 | Calculation of apparent sensitivity (aSe) for each PCR system of the Htrt PCR assay.** Mixtures of positive control DNAs were tested in 12 replicates for 4 dilution ranges. aSe is calculated as the ratio of the number of positive replicates to the total number of replicates. The expected Ct using classical real time PCR is shown for each dilution range.
